# Effects of inhaling *Cunninghamia lanceolata* essential oil on the physiological and psychological relaxation of university students

**DOI:** 10.3389/fpsyg.2025.1638492

**Published:** 2025-09-24

**Authors:** Jing Liu, Meng Liu, Liang Peng, Huanzhu He, Ruijie Sun, Weiyin Chang

**Affiliations:** ^1^College of Forestry, Fujian Agriculture and Forestry University, Fuzhou, China; ^2^Department of International Tea Industry and Culture, Pusan National University, Gyeongnam, Republic of Korea; ^3^Laboratory of Virtual Teaching and Research on Forest Therapy Specialty of Taiwan Strait, Fujian Agriculture and Forestry University, Fuzhou, China; ^4^Cross-Strait Research Center for Forest Therapy and Environmental Education, Fujian Agriculture and Forestry University, Fuzhou, China

**Keywords:** *Cunninghamia lanceolata*, essential oil, aromatherapy, electroencephalogram, natural therapy, health benefits

## Abstract

University students face multiple stressors, which can impair their physical and mental health without timely intervention. Despite the close link between nature and wellbeing, as well as the growing interest in using natural substances for health management, the effectiveness of *Cunninghamia lanceolata* essential oil (*C. lanceolata* EO) in alleviating emotional disorders among university students remains underexplored. This study investigated the physiological and psychological effects of inhaling *C. lanceolata* EO among 40 healthy university students (mean age: 21.75 ± 1.82 years). Subjects inhaled room air (control) and *C. lanceolata* EO for 5 min each. Electroencephalograms (EEG), heart rate variability (HRV), blood pressure (BP), and the Profile of Mood States (POMS) were used as assessment indicators. The findings showed that, at the physiological level, compared with inhaling room air (control), the mean theta (*θ*) wave in the frontal and parietal lobes (*p* < 0.05) and the mean alpha (*α*) wave in the whole brain (*p* < 0.001) were significantly higher during inhalation of *C. lanceolata* EO. Simultaneously, the mean beta (*β*) wave in the frontal, temporal, and parietal lobes was significantly lower (*p* < 0.05); stress-related indices, including heart rate (HR), BP, low-frequency power (LF), and the LF/HF ratio, were significantly lower (*p* < 0.01), while the Standard Deviation of Normal-to-Normal intervals (SDNN) and high-frequency power (HF) were significantly higher (*p* < 0.01), indicating a more relaxed physiological state. Psychologically, during inhalation of *C. lanceolata* EO, scores for negative emotions (tension, depression, and fatigue) and total emotional disturbance (TMD) on the POMS were significantly lower (*p* < 0.05), while scores for positive emotions (energy and self-esteem) were significantly higher (*p* < 0.01). These results suggest that inhaling *C. lanceolata* EO may have a relaxing effect on the physiological and psychological states of university students, indicating that inhaling *C. lanceolata* EO has a positive impact on promoting their mental and physical relaxation. Future long-term empirical studies could be conducted to further explore the actual stress-relieving effects of *C. lanceolata* EO.

## 1 Introduction

University students are in a unique transitional period of their lives. They face stressors from various aspects, such as academics, the economy, social life, employment, and family (Elena et al., n.d.). Excessive stress can reduce quality of life, happiness, and life satisfaction (김남정 and Lim, 2012), and may lead to common emotional symptoms such as anxiety and depression (Amaro et al., 2024). Furthermore, severe emotional disorders can affect physical and mental health, potentially leading to suicidal thoughts (Duffy et al., 2019). Therefore, there is an urgent need to develop simple, effective physical and mental health programs that address the specific needs of university students.

Among the various methods of physical and mental regulation, aromatherapy has been proven to significantly reduce anxiety, improve sleep quality, and relieve pain when used as an adjuvant therapy (Hedigan et al., 2023; Yin et al., 2024; Akbas Uysal et al., 2024). The primary methods of aromatherapy are inhalation, oral administration, massage, and bathing, all of which help prevent the onset of, alleviate, or treat psychological and emotional disorders (Farrar and Farrar, 2020). One common form of aromatherapy is inhalation, in which aromatic particles enter the body through the nasal cavity and stimulate olfactory receptors. These signals are then transmitted to the limbic system, which triggers emotional responses such as pleasure, relaxation, and instinctive behaviors. The limbic system consists of the amygdala, hippocampus, cingulate gyrus, hypothalamus, and other neural structures in the brain. These structures are primarily involved in important physical and psychological activities such as emotion regulation, memory formation, and sleep cycle regulation (Kremen et al., 2025). The limbic system also acts on the autonomic nervous system, regulating physiological processes such as heart rate, blood pressure, respiratory rate, memory, and stress responses (Florian et al., 2025).

Essential oils (EOs) are complex, volatile mixtures extracted from aromatic plants that usually contain ketones, aldehydes, and aromatic compounds (Vora et al., 2024). Numerous studies have demonstrated the efficacy of EOs in alleviating anxiety, regulating negative emotions, and enhancing sleep quality in various populations, including women (Moon et al., 2020), drivers (Hu et al., 2024), teachers (Liu et al., 2013). In particular, a growing number of studies have consistently shown positive effects of aromatherapy on university students. For instance, *lavender* EO reduced anxiety levels in freshmen with social anxiety during test evaluations (Solomon et al., 2024); *bergamot* EO improved sleep quality by promoting psychophysiological relaxation (Wakui et al., 2023); Black pepper EO may alleviate stress in university students by inhibiting excessive sympathetic nervous system (SNS) excitation and promoting local blood flow (Dilrukshi et al., 2024).

However, many studies have also shown that exposure to scents does not always reduce stress or anxiety. For example, one study found that lower doses of lavender EO had an anti-anxiety effect, while higher doses were comparable to the control group and did not show significant differences, suggesting that we need to precisely control the appropriate dosage (Angulo et al., 2025). Another study validated the auxiliary effects of EOs in progressive muscle relaxation (PMR) training, and results showed that regardless of whether scented nose clips were used, all subjects experienced a significant decrease in heart rate after PMR training (*p* < 0.001), but there was no significant difference between the scented group and the non-scent group (*Fs* < 1; F-statistic from ANOVA), and scent also did not alleviate stress during the second phase of the stress task. Possible reasons for the lack of significant results include: some subjects in the no-scent group may have been psychologically influenced by being told that the “scent concentration was extremely low”; subjects selected their own scents (e.g., lavender was the most common), and individual differences may have diluted the overall effect; the experimental task (word association) did not elicit sufficient anxiety or fear. Additionally, since subjective anxiety scores were not recorded, relying solely on heart rate metrics may have obscured differences in emotional experiences (Mahmut et al., 2023). Another study found that subjects who received the expectation cue showed significantly shorter reaction times on cognitive tasks under stress conditions, along with reduced N200/P300 latency (indicating more efficient cognitive function). However, the lavender group and the control group showed no significant differences across all measured indicators, suggesting that lavender aroma did not influence cognitive task performance under stress conditions. Possible reasons for the lack of significant aromatic effects include: small effect sizes, which require larger sample sizes to detect; cognitive tasks being too simple to reach the effect detection threshold; task duration inducing fatigue effects; issues with the method of EO exposure (e.g., the dose of 3 drops of EO may be insufficient, and the simple cotton pad application method may not ensure consistent exposure); failure to activate specific neural pathways involved in aroma effects; and differences in the nature of experimental stress (cognitive tasks) versus clinical stress (e.g., medical settings) (Chamine and Oken, 2015).

Furthermore, existing research on the health benefits of EOs mostly focuses on flowers (Pourshaikhian et al., 2024; Genc and Saritas, 2020), herbs (Lucena et al., 2024; Mank-halati et al., 2024), and shrubs (Bozova et al., 2024; Doner et al., 2024; Goncalves et al., 2024). There are relatively few studies on EOs from ligneous angiosperms, especially *C. lanceolata*. Additionally, the evaluation indicators in existing research on EOs of ligneous angiosperms are not comprehensive. For example, a study investigating the effects of Japanese cedar EO on improving employee mental health included only 9 male subjects in its sample, which is limited by its small sample size and lack of gender diversity. In terms of physiological indicator testing, only saliva stress markers were analyzed, and important stress-related indicators such as autonomic nervous system function were not covered (Matsubara et al., 2017).

To more scientifically and accurately explore the regulatory mechanism of aromatherapy on emotions, electroencephalography (EEG) technology has become an important research tool. Because odor is closely related to emotions, the impact of aromatic odors on brain activity can be measured quantitatively through EEG (Kato et al., 2022). EEG records electrical signals emitted by the brain, which in turn reflect its real-time activity (Fox et al., 2024). The power spectrum is divided into distinct frequency bands, including delta (*δ*) wave (0.5–4 Hz), theta (*θ*) wave (4–8 Hz), alpha (*α*) wave (8–13 Hz), beta (*β*) wave (13–30 Hz), and gamma (*γ*) wave (30–50 Hz). Each frequency band corresponds to a different brain state (Sowndhararajan and Kim, 2016). For instance, theta wave is associated with cognition and memory, as well as with deep relaxation (Jacobs et al., 1996), while *α* wave indicates a relaxed, quiet, and awake state (Grassini et al., 2022). *β* wave, on the other hand, reflects a tense state of brain activity and is commonly found in an alert state (Lee et al., 2014). These brain waves play a key role in emotional regulation and cognitive processing. Changes in their EEG signals can reveal the intervention mechanism of EOs and evaluate their anxiolytic and relaxing effects. Beyond frequency bands, EEG also enables analysis of brain activity across different anatomical regions. EEG regional analysis focuses on four major lobes: the frontal, temporal, parietal, and occipital lobes, which govern emotional regulation, olfactory perception, somatosensory processing, and visual integration, respectively (Juran et al., 2016; Maksimenko et al., 2018; Nickel and Seitz, 2005; Nejati et al., 2021). Based on data from the central and autonomic nervous systems, EEG can reliably and objectively analyze subtle emotional changes. Previous studies have used EEG to explore the potential therapeutic value of various plant EOs. For instance, a study has used EEG to demonstrate that exposure to peppermint, rose, and lavender EOs can improve subjective perception and enhance positive emotions (Zhang et al., 2025). Additionally, EEG studies on the relaxing effects of lavender EO have indicated that *θ* and *α* waves associated with relaxation significantly increased in subjects’ frontal lobes (Afghan et al., 2024). Furthermore, other studies have used EEG to investigate the effects of *Cinnamomum camphora* EO on human emotional states, demonstrating that *C. camphora* EO can promote cognitive processes such as memory consolidation and retention while improving emotional states (Gong et al., 2024). EEG can accurately reflect functional differences in frequency bands and brain regions, and it has demonstrated significant value in researching the effects of plant EOs.

*Cunninghamia lanceolata* (Lamb.) Hook., a common coniferous tree species in southern China, belongs to the evergreen genus of the Cupressaceae (Cui and Matsumura, 2020). Its EO contains various volatile organic compounds with biological activity, including terpenoids, alcohols, naphthalenes, and terpenoid esters (Xin et al., 2012; Su et al., 2012; Li et al., 2020; Kuspradini et al., 2016). Research has confirmed that *C. lanceolata* EO has anti-inflammatory and analgesic effects (Su et al., 2012; Xin et al., 2012). The main component, *α*-cedrene, has antibacterial and antioxidant properties (Usman et al., 2024; Bellioua et al., 2024). Cedrol exhibits antibacterial, anti-inflammatory, and neuroprotective properties (Forouzanfar et al., 2025; Asgharzade et al., 2025; Rastegar-Moghaddam et al., 2024), and *β*-cedrene inhibits the growth of human intestinal bacteria (Kim and Lee, 2015). These findings demonstrate the high medicinal value of *C. lanceolata* EO. Previous studies have revealed the regulatory effects of Cupressaceae EOs on emotional and physiological states. Specifically, an earlier study experimentally found that when subjects inhaled hinoki EO (a species of *Cupressaceae*), changes occurred in blood pressure (BP) and heart rate (HR), with decreased sympathetic nerve activity and increased parasympathetic nerve activity. These results suggested that *Cupressaceae* EOs may improve emotional states (Chen et al., 2015). Subsequently, a comparative study determined that inhaling *Cupressaceae* EOs was more effective in reducing tension and depression and providing a sense of natural comfort than Pinaceae EOs (Gho et al., 2023). Meanwhile, studies on workplace settings further confirmed that using *Cupressaceae* EOs in office environments can help relieve employees’ stress and promote their physical and mental health (Matsubara et al., 2023). These studies on *Cupressaceae* EOs imply that *C. lanceolata* EO, as a member of this family, may share similar mood-regulating effects. However, research on the effect of *C. lanceolata* EO on human emotions is limited, and its therapeutic potential for emotional health remains underexplored.

In conclusion, given the limited research on the emotional regulatory effects of *C. lanceolata* EO and the lack of comprehensive evaluation metrics in previous studies on woody plant EOs, this study aims to investigate the potential effects of *C. lanceolata* EO on physiological and psychological relaxation in university students from a multidimensional perspective, using EEG, heart rate variability (HRV), BP, and the Profile of Mood States (POMS) psychological questionnaire.

To achieve this objective, we compared the physiological and psychological indicators of university students during inhalation of *C. lanceolata* EO and room air under daily stress conditions, and discussed the findings in conjunction with indicator definitions, trend implications, and previous conclusions. Additionally, we improved the research methodology to address issues identified in previous studies: subjects were not informed of any information regarding the scent beforehand to avoid psychological expectations influencing the experimental results; a standardized diffusion device was used to optimize the aroma exposure method, ensuring consistency in exposure; we employed a within-subject experimental design to reduce individual trait differences with self as control; we also eliminated fatigue differences caused by temporal sequence; and we did not artificially impose additional stress, instead we conducted it under the normal stress conditions of university students. The study also expanded the measurement indicators for a multidimensional quantitative analysis of the potential benefits of *C. lanceolata* EO.

The relevant research hypotheses are shown below (all compared with inhalation of room air (control)):

*Hypothesis 1:* Physiological recovery.

*H1a:* During inhalation of *C. lanceolata* EO, the subjects’ mean *θ* and mean *α* waves will be significantly higher, while the mean *β* wave will be significantly lower.

*H1b:* During inhalation of *C. lanceolata* EO, the subjects’ HR, low-frequency power (LF), and LF/HF ratio will be significantly lower, while Normal-to-Normal intervals (SDNN) and HF will be significantly higher.

*H1c:* During inhalation of *C. lanceolata* EO, the subjects’ systolic blood pressure (SBP) and diastolic blood pressure (DBP) will be significantly lower.

*Hypothesis 2:* Psychological recovery.

*H2a:* During inhalation of *C. lanceolata* EO, the subjects’ negative emotion indicators (tension, depression, anger, fatigue, and panic) will be significantly lower, while the positive emotion indicators (energy and self-esteem) will be significantly higher.

*H2b:* During inhalation of *C. lanceolata* EO, the subjects will have significantly lower total mood disturbance (TMD) scores.

## 2 Materials and methods

### 2.1 Subjects

This study recruited 41 volunteers from Fujian Agriculture and Forestry University. Their ages ranged from 18 to 26 years. However, due to technical issues with the equipment, data from one subject could not be successfully collected, resulting in a final valid sample of 40 subjects, with 22 females and 18 males. All subjects were university students who were about to take exams within a week, had no cardiovascular diseases, neuropsychiatric symptoms, or nasal diseases, and did not experience any physical discomfort. They also did not have any allergic reactions to EOs. Subjects were required to avoid wearing perfume and consuming food, beverages, or medications that might affect the study results for 24 h before the experiment. They were also required to keep their scalps clean and dry. Before the experiment began, subjects were informed of the purpose of the study and its duration. After fully understanding the experimental procedures, all subjects voluntarily signed a written informed consent form. This study was approved by the Human Research Ethics Committee of Fujian Provincial Hospital (K2019-03-006).

### 2.2 Olfactory stimulation

**Figure 1:**
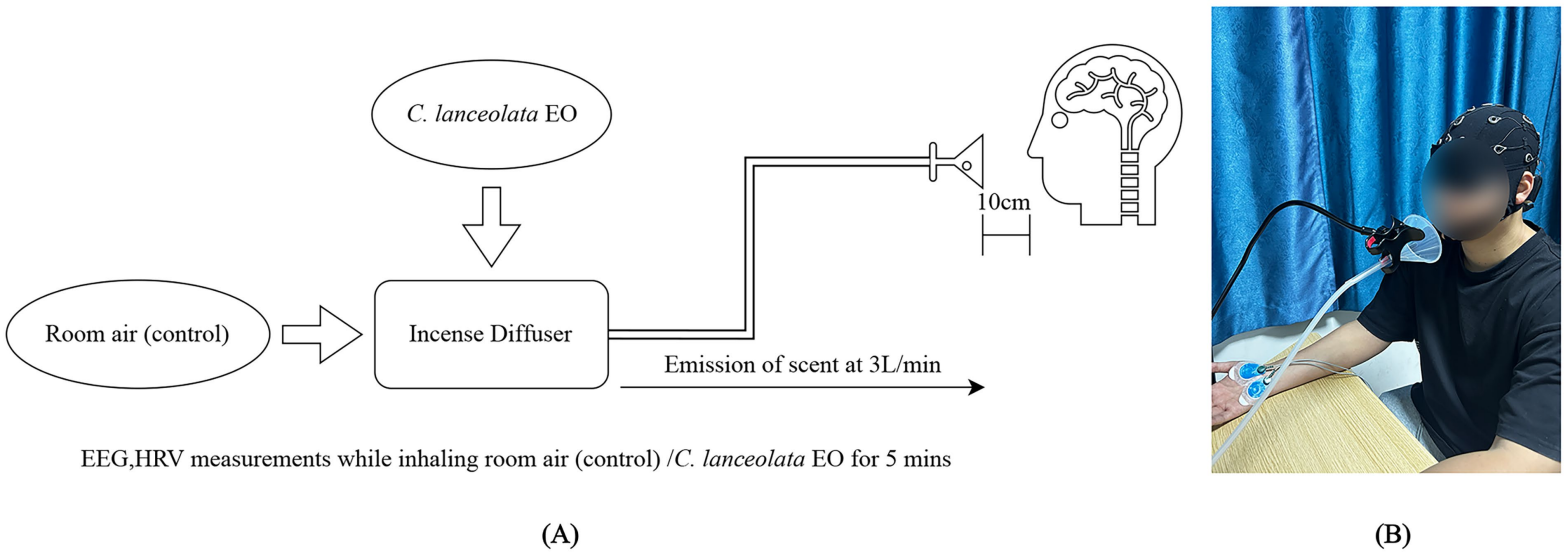
Specification of the experimental setup **(A)** Aromatherapy diffuser; **(B)** Experimental scene.

The *C. lanceolata* EO used in this study was provided by the Department of Wood-Based Materials and Design, National Chiayi University. The oil was extracted from *C. lanceolata* leaves by steam distillation and diluted to a concentration of 3% using dipropylene glycol as a solvent. The diluted *C. lanceolata* EO was then atomized and released through an aromatherapy diffuser at a flow rate of 3 L/min for 5 min. This ensured the stability and controllability of the EO release concentration (Figure 1A). The experimental environment was kept at a constant temperature of 25 °C and relative humidity of 50%. The experimental room measured 4 × 3 × 2.5 m. Subjects smelled the odor through a tube connected to a funnel-shaped dispenser installed on the table. The funnel was 10 cm from the subjects’ noses. The device was designed to diffuse the odor evenly and maintain a relatively stable stimulation intensity. This effectively reduced the interference of external factors, such as airflow and distance, on the experimental results. Before the experiment began, the subjects were not informed of the specific odor. They were also blindfolded to avoid influencing the results by subjective expectations.

### 2.3 Experimental design

**Figure 2:**
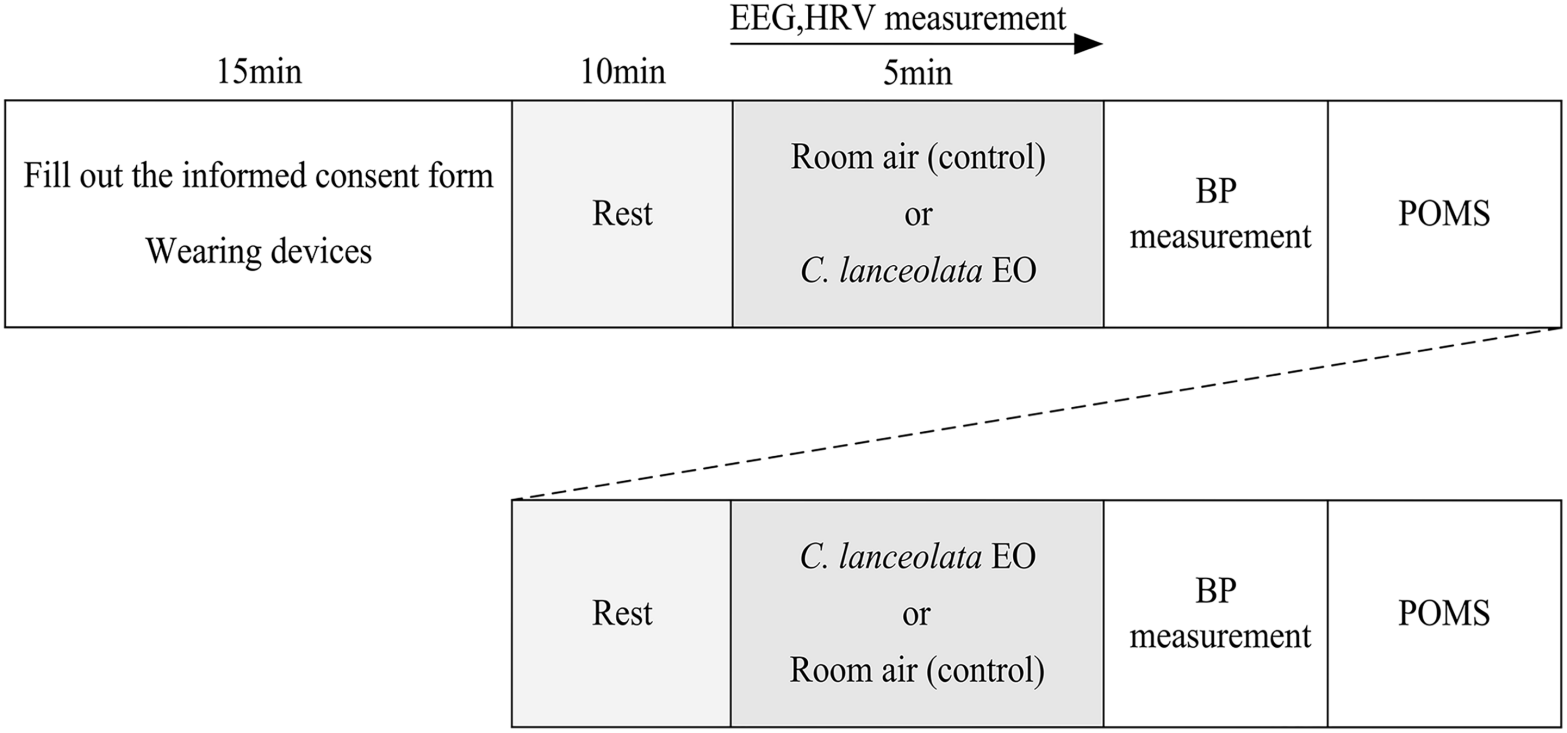
Experimental procedure.

This study adopted a within-subject experimental design. Each subject used room air as a control and compared differences in physiological and psychological indicators between inhaling room air and inhaling *C. lanceolata EO* under normal stress conditions to infer the effects of *C. lanceolata* EO on the physiology and psychology of university students. To minimize the effects of sequence and fatigue, approximately half of the subjects first inhaled room air (control) for 5 min, and then inhaled *C. lanceolata* EO for 5 min. The remaining subjects first inhaled *C. lanceolata* EO for 5 min, then inhaled room air (control) for 5 min. A 10-min rest interval was set between the two odor exposures to ensure that the subjects were in as homogeneous a state as possible before inhaling each odor. This design effectively controls for individual variability and is suitable for observing differences in the same group of subjects under different intervention conditions. Figure 2 shows the experimental scheme (the experimental sequence is outlined therein), and the specific implementation is as follows:

After entering the experimental environment, the subjects were informed of the experimental procedures and purpose. They filled out and signed the informed consent form, and the experimenters assisted them in wearing the physiological monitoring equipment, which took about 15 min. Then, the subjects were given 10 min to rest and adapt to reach a stable physiological and psychological state. After resting, the subjects inhaled either room air or *C. lanceolata* EO for 5 min. Then, after another 10 min of rest, they inhaled the other odor. While inhaling the gas, their physiological data, such as Electroencephalograms (EEG) and HRV were continuously collected. After inhaling each gas, BP data were immediately collected and subjects completed the POMS psychological questionnaire shortly thereafter. After the experiment, the physiological monitoring equipment was removed, and the entire experimental session lasted about 60 min.

### 2.4 GC–MS analysis of *C. lanceolata* EO

The volatile aromatic components of *C. lanceolata* EO were identified using gas chromatography–mass spectrometry (GC–MS). The analysis was performed using a YLSZJ-SB-258 GC–MS system equipped with an HP-5MS quartz capillary column (30 m × 0.25 mm × 0.25 μm). The injector temperature was set at 250 °C, and the initial column temperature was maintained at 60 °C for 2 min, followed by an increase to 200 °C at a rate of 3 °C/min, where it was held for 15 min. The injection volume was 1 μL, and helium was used as the carrier gas at a flow rate of 1 mL/min and a pressure of 0.15 MPa. The injector and ion source temperatures were set at 220 °C and 230 °C, respectively. The components were analyzed qualitatively and quantitatively by comparing their mass spectra with the National Institute of Standards and Technology (NIST) library. The relative percentages of the components were quantified via area normalization. A total of 16 major components were identified with similarity matches greater than 90%, accounting for approximately 69% of the total composition (Table 1).

**Table 1:** The main volatile organic compounds of *C. lanceolata* EO.

S. number	Component	Retention time (min)	Formula	Peak area (%)
1	*α*-Cedrene	33.60	C₁₅H₂₄	19.04
2	Cedrol	38.49	C₁₅H₂₆O	15.65
3	*β*-Cedrene	33.81	C₁₅H₂₄	6.86
4	n-Hexadecanoic acid	44.29	C₁₆H₃₂O₂	4.04
5	cis-Thujopsene	34.01	C₁₅H₂₄	3.00
6	*β*-Elemene	32.55	C₁₅H₂₄	2.84
7	*α*-Terpinol	26.60	C₁₀H₁₈O	2.63
8	*α*-Alaskene	35.93	C₁₅H₂₄	2.48
9	*β*-Selinene	35.46	C₁₅H₂₄	2.41
10	*δ*-Cadinene	36.043	C₁₅H₂₄	2.34
11	*β*-Copaene	35.193	C₁₅H₂₄	1.78
12	Di-epi-*α*-cedrene-(I)	32.624	C₁₅H₂₄	1.74
13	Cedryl acetate	41.089	C₁_7_H_2_₈O_2_	1.59
14	Octadecanoic acid	48.172	C₁_8_H_36_O_2_	1.41
15	*α*-Selinene	35.612	C₁_5_H_24_	1.19
16	*α*-Pinene	15.491	C_10_H_16_	0.76

### 2.5 Physiological measurements

#### 2.5.1 Electroencephalogram recording

This study used the Smarting Mobi portable EEG system (developed by mBrainTrain) to monitor subjects’ brain activity in real time. The study focused on the *α*, *β*, and *θ* frequency bands to evaluate the potential effects of aromatherapy on brain function (Figure 1B). The EEG system’s sampling frequency was set to 500 Hz, and the electrodes were placed precisely at specific positions according to the international 10–20 system. These positions included Fp1, Fp2, F3, F4, F7, F8, C3, C4, T7, T8, P3, P4, P7, P8, O1, O2, AFz, Fz, Cz, CPz, Pz, and POz. The reference electrodes were positioned at M1 and M2. During the experiment, GT5 medical EEG gel (produced by Wuhan GreenTech Co., Ltd., China) was injected into the electrode holes of the EEG cap. This ensured that the contact impedance between the scalp and each electrode remained below 5 kΩ, thereby allowing for the acquisition of high-quality signals.

To improve data quality, the original EEG signals collected from the scalp electrodes were first filtered using MATLAB R2013b to eliminate electrical artifacts caused by blinking or body movement. Second, the fast Fourier transform (FFT) was applied to convert the time-domain signals into frequency-domain signals. EEGLAB (running on MATLAB R2013b) software was then used to generate brain topographic maps, which intuitively compared the spatial distribution differences in the mean activity energy of whole-brain *α*, *β*, and *θ* waves during inhalation of room air (control) and *C. lanceolata* EO. This visualization method helps to analyze the effects of *C. lanceolata* EO on the activities of different brain regions from a spatial perspective, thereby providing a visual basis for exploring its anxiolytic and relaxing mechanisms.

#### 2.5.2 Heart rate variability and blood pressure

In this study, a Mindware Mobile Instrument (produced by MindWare Technologies LTD.) was used to collect electrocardiogram (ECG) data focusing on heart rate variability (HRV). HRV software (RHMS-10-A22B; YoujiaLi Information Technology Co., Ltd., Jinan, China) was used to generate reports. HRV is a sensitive indicator of changes in heart rhythm and is widely recognized as a reliable marker of autonomic nervous system (ANS) activity (Carandina et al., 2021). It has been applied in multiple disciplines, such as psychology, medicine, and sports science (Perna et al., 2020). HRV was analyzed using the following key indicators: heart rate (HR), standard deviation of NN intervals (SDNN), low frequency (LF, 0.04–0.15 Hz), high frequency (HF, 0.15–0.4 Hz), and LF/HF ratio. HR reflects the frequency of heartbeats, while SDNN measures the overall variability of cardiac cycles. In frequency-domain analysis, LF and HF, respectively indicate the activities of the sympathetic and parasympathetic nervous systems (Pham et al., 2021), and the LF/HF ratio reflects ANS regulation balance. An increase in HR is typically indicative of anxiety and stress, whereas a decrease may suggest relaxation (Hongratanaworakit and Buchbauer, 2005). Higher SDNN reflects increased parasympathetic activity and decreased sympathetic activity (Bogdan et al., 2024). Increased HF activity indicates enhanced parasympathetic nerve activity, often associated with relaxation, whereas elevated LF activity correlates with heightened sympathetic activity, linked to arousal, tension, or anxiety (Yamashita et al., 2021). Fluctuations in the LF/HF ratio reveal the balance between sympathetic and parasympathetic activities. Decreased HR variability is associated with anxiety and depressive states (Dell’Acqua et al., 2020).

In addition to HRV, BP was also measured as an indicator of ANS regulation, as the autonomic nerves play a critical role in BP modulation (Tomitani et al., 2021). BP measurements complement HRV analysis in assessing ANS function. BP was recorded using a monitor (HEM-92200 L, Omron Dalian Co., Ltd.). BP is divided into systolic blood pressure (SBP), which reflects the maximum arterial pressure during ventricular contraction, and diastolic blood pressure (DBP), representing the minimum pressure during ventricular diastole. The BP is significantly higher in negative emotional states than in positive ones, indicating that emotions consistently and universally impact BP (Shahimi et al., 2022). Changes in these indicators effectively reflect the ANS’s response to emotions and stress. This thereby provides an objective basis for evaluating the relaxing effect of *C. lanceolata* EO in this study.

### 2.6 Psychological measurement

This study used the Profile of Mood States (POMS), one of the most widely used emotional psychological measurement tools in environmental psychology, to measure the psychological effects of olfactory stimulation (Albani et al., 2005). Initially developed by Grove and Prapavessis (1992) and later revised by Zhu (1995), the scale was adapted to establish a norm suitable for the Chinese population (Zhu, 1995). The 40-item questionnaire covers seven emotional dimensions: five negative states (“tension,” “depression,” “anger,” “fatigue,” and “panic”) and two positive states (“energy” and “self-esteem”). Each item is scored using a seven-point Likert scale, a commonly used method for quantifying subjects’ responses (Dolan, 1994). The Total Mood Disturbance (TMD) score is calculated as: (tension + depression + anger + fatigue + panic) − (energy + self-esteem) + 100 (Zhu, 1995). A higher TMD score indicates a more negative emotional state (Watanabe et al., 2024), and a lower TMD score reflects a more positive emotional state characterized by decreased stress and anxiety levels and increased calmness and relaxation (Grove and Prapavessis, 1992). According to psychological research standards, a Cronbach’s *α* value greater than 0.7 indicates acceptable reliability (0.8–0.9 is considered good) (Cronbach, 1951). In this study, Cronbach’s *α* was used to evaluate the internal consistency of each dimension in the two gas environments. After inhaling room air, the Cronbach’s *α* values were 0.797, 0.766, 0.874, 0.756, 0.915, 0.877, and 0.827. After inhaling *C. lanceolata* EO, the Cronbach’s *α* values were 0.918, 0.788, 0.718, 0.837, 0.906, 0.841, and 0.799. The *α* values of each psychological index in each dimension during inhalation of room air or *C. lanceolata* EO ranged from 0.718 to 0.918, exceeding the acceptable threshold and demonstrating good stability. These results confirm that the scale has ideal reliability and internal consistency, making it suitable for subsequent research.

### 2.7 Data analysis

In this study, EEG signals from the entire brain were systematically preprocessed. The time series of these signals were then segmented according to the experimental protocol to ensure consistent and accurate data processing. Subsequently, frequency-domain features of alpha (8–13 Hz), beta (13–30 Hz), and theta (4–8 Hz) waves were extracted from each subject’s electrode channels, focusing on EEG power in the frontal, temporal, parietal, and occipital lobes. Thereafter, the average power spectral density of these frequency bands in the selected brain regions was calculated. Brain topographic maps were then constructed using EEGLAB R2013b software to visually present the energy distribution of the different frequency bands.

The statistical data of the subjects, including EEG (*α*, *β*, *θ* waves), HRV (HR, SDNN, LF, HF, and LF/HF ratio), BP (SBP, DBP), and POMS scores, were analyzed using SPSS Statistics 27. Descriptive statistics, such as mean (M) and standard deviation (SD), were reported. To evaluate the differences between room air and *C. lanceolata* EO inhalation conditions, we conducted paired t-tests at a significance level of *α* = 0.05 (two-tailed). Cohen’s *d* was calculated for each dimension to quantify the effect size (*d* = 0.2 [small]; *d* = 0.5 [medium]; *d* = 0.8 [large]). Following the statistical analysis, GraphPad Prism 9 was used to visualize the results.

## 3 Results

### 3.1 Physiological responses

#### 3.1.1 Comparison of differences in EEG topography maps during inhalation of room air (control) and *C. lanceolata* EO

**Figure 3:**
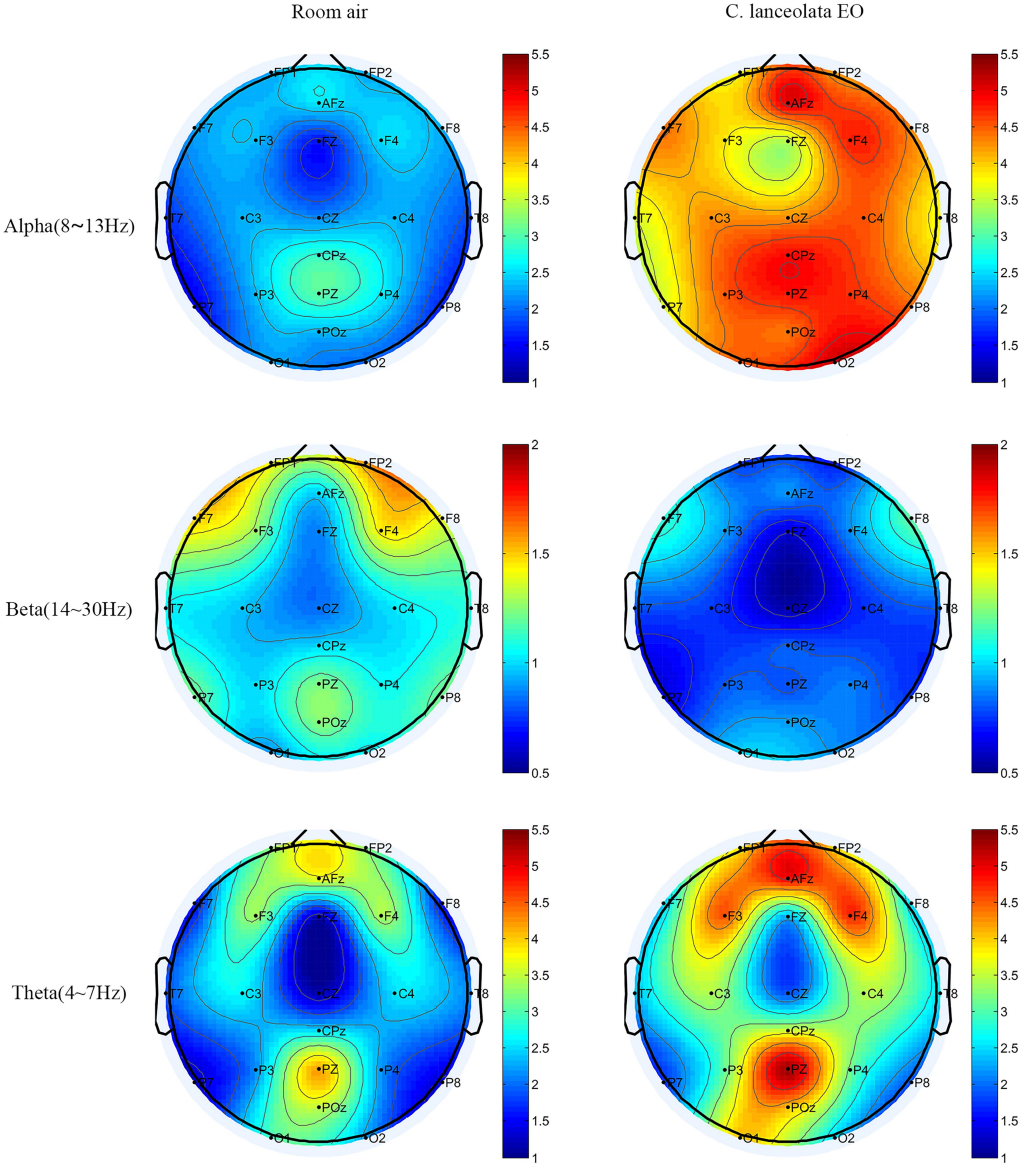
The t-mapping of EEG power spectrum changes during inhalation of room air and *C. lanceolata* EO.

Figure 3 shows the visual presentation of the spatial distribution of average *α*, *β*, and *θ* wave values in the whole brain when inhaling room air and *C. lanceolata* EO. The top, middle, bottom, and sides of the brain topography map correspond to the frontal lobe, parietal lobe, occipital lobe, and temporal lobe, respectively. Red represents higher values and blue represents lower values. The results showed that, compared to room air (control), inhalation of *C. lanceolata* EO was associated with significantly higher mean *θ* wave (4–8 Hz) in the frontal and parietal lobes and higher whole-brain mean *α* wave (8–13 Hz). In contrast, mean *β* wave (13–30 Hz) was significantly lower in the frontal, temporal, and parietal lobes. Additionally, the topography clearly showed that the *α* wave was significantly higher in the right-biased frontal lobe during inhalation of *C. lanceolata* EO, consistent with previous findings that “left-biased frontal *α* wave is positively correlated with depression severity, while right-biased frontal *α* wave is negatively correlated with depression severity” (Kopanska et al., 2025). These results suggest that inhaling *C. lanceolata* EO may influence activity in different brain regions, with *θ* and *α* wave power (linked to relaxation) and *β* wave power (linked to tension) contributing to differences in the EEG spectrum that reflect a potential relaxing effect.

#### 3.1.2 Multiband EEG power spectrum

**Figure 4:**
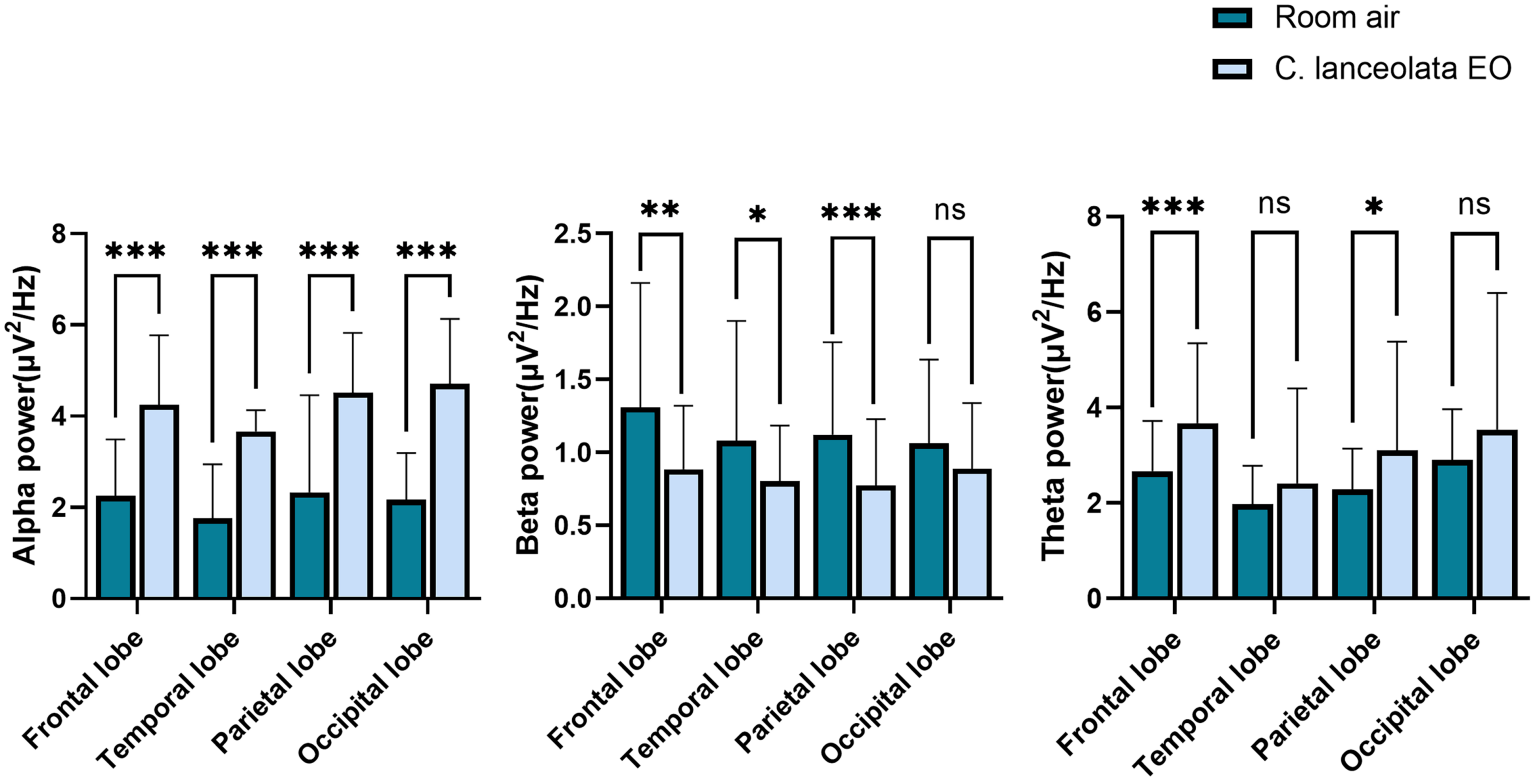
Paired t-test results for EEG power spectra changes during inhalation of room air and *C. lanceolata* EO.

Paired-samples t-tests were performed to compare EEG differences during inhalation of room air (control) and *C. lanceolata* EO (Figure 4), and these results are summarized in Table 2. During inhalation of *C. lanceolata* EO, the alpha (*α*) wave power in all brain regions was significantly higher than that during inhalation of room air (control): frontal lobe 2.00 μV^2^/Hz (*p* < 0.001), temporal lobe 1.90 μV^2^/Hz (*p* < 0.001), parietal lobe 2.19 μV^2^/Hz (*p* < 0.001), occipital lobe 2.54 μV^2^/Hz (*p* < 0.001). In contrast, the beta (*β*) wave power in the frontal, temporal, and parietal lobes was significantly lower than that during inhalation of room air (control): frontal lobe 0.42 μV^2^/Hz (*p* < 0.01), temporal lobe 0.28 μV^2^/Hz (*p* < 0.05), parietal lobe 0.35 μV^2^/Hz (*p* < 0.001). The theta (*θ*) wave power in the frontal and parietal lobes was significantly higher than that during inhalation of room air (control): frontal lobe 1.01 μV^2^/Hz (*p* < 0.001), parietal lobe 0.85 μV^2^/Hz (*p* < 0.05). These results suggest that inhalation of *C. lanceolata* EO affects brain activity, mainly by enhancing *α* and *θ* wave activities throughout the brain and suppressing *β* wave activities in the frontal and temporal lobes.

**Table 2:** Paired t-test results of the EEG power spectrum during inhalation of room air (control) and *C. lanceolata* EO.

Variables (Unit)	Site	Air		EO		t-test	*p*-value	Cohen’s *d*
		Mean	SD	Mean	SD			
Alpha (μv^2^/Hz)	FL	2.25	1.24	4.25	1.52	−5.91	0.000***	−0.93
	TL	1.76	1.18	3.66	0.47	−9.02	0.000***	−1.43
	PL	2.33	2.13	4.52	1.31	−5.06	0.000***	−0.80
	OL	2.17	1.02	4.71	1.42	−8.78	0.000***	−1.39
Beta (μv^2^/Hz)	FL	1.31	0.85	0.88	0.43	3.63	0.001**	0.57
	TL	1.08	0.82	0.80	0.38	2.30	0.027*	0.36
	PL	1.12	0.63	0.77	0.45	4.88	0.000***	0.77
	OL	1.06	0.57	0.89	0.45	1.78	0.084	0.28
Theta (μv^2^/Hz)	FL	2.66	1.06	3.67	1.67	−4.26	0.000***	−0.67
	TL	1.98	0.80	2.21	1.99	−0.70	0.490	−0.11
	PL	2.27	0.86	3.13	2.26	−2.57	0.014*	−0.41
	OL	2.89	1.07	3.03	2.86	−0.34	0.732	−0.05

Air, room air; EO, *C. lanceolata* EO; FL, frontal lobe; TL, temporal lobe; PL, parietal lobe; OL, occipital lobe. *N* = 40; values are represented as mean ± SD. Significant differences: ns (not significant); *p < 0.05; **p < 0.01; ***p < 0.001.

#### 3.1.3 Heart rate variability and hemodynamic responses

**Figure 5:**
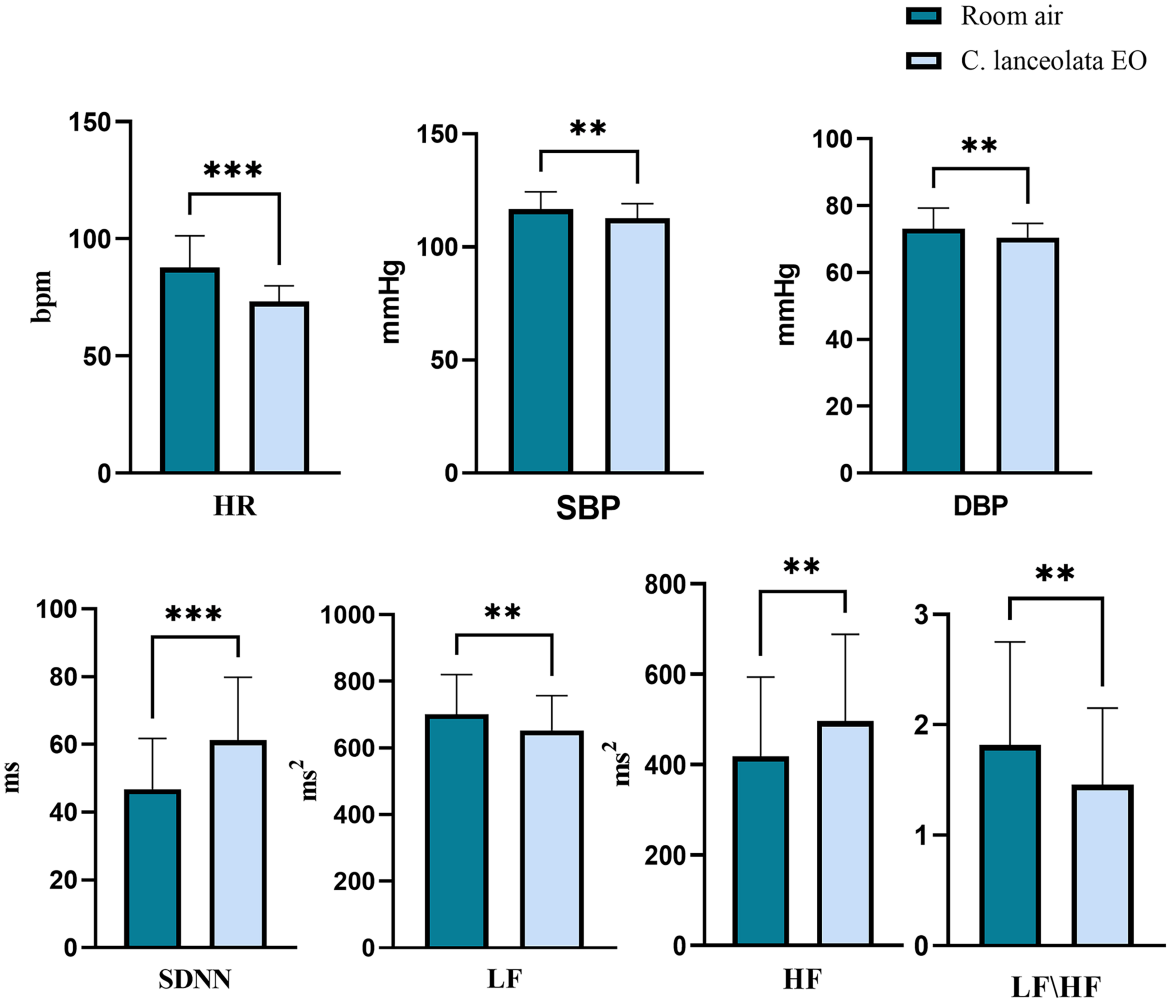
Paired t-test results for heart rate variability (HRV) and blood pressure (BP) during inhalation of room air (control) and *C. lanceolata* EO.

Paired-samples t-tests were conducted to evaluate significant differences in HRV and BP during inhalation of room air (control) versus *C. lanceolata* EO (Figure 5), with results summarized in Table 3. This analysis evaluated statistically significant differences between mean HR, SDNN, LF, HF, and LF/HF ratio during room air (control) inhalation versus *C. lanceolata* EO inhalation. The results showed that stress indicators during inhalation of *C. lanceolata* EO were significantly lower than those during inhalation of room air (control), while indicators related to relaxation were significantly higher. Specifically, compared with inhalation of room air (control), under the *C. lanceolata* EO inhalation condition, the HR of the subjects was significantly lower by 14.47 beats/min (*p* < 0.001). The mean SDNN was significantly higher by 14.48 ms (*p* < 0.001), the mean LF was significantly lower by 47.86 ms^2^ (*p* < 0.01), the mean HF was significantly higher by 78.05 ms^2^ (*p* < 0.01), and the mean LF/HF was significantly lower by 0.36 (*p* < 0.01). Additionally, the mean SBP was significantly lower by 4.0 mmHg (*p* < 0.01), and the mean DBP was significantly lower by 2.7 mmHg (*p* < 0.01). These results suggest that inhaling *C. lanceolata* EO can significantly improve heart rate variability and increase parasympathetic nerve activity while reducing sympathetic nerve activity.

**Table 3:** Paired t-test of HRV and blood pressure (BP) during inhalation of indoor air (control) and *C. lanceolata* EO.

Variables (Unit)	Air		EO		t-test	*p*-value	Cohen’s *d*
	Mean	SD	Mean	SD			
HR (bmp)	87.64	13.64	73.18	6.74	8.63	0.000***	1.37
SBP (mmHg)	116.63	7.84	112.63	6.50	3.32	0.002**	0.53
DBP (mmHg)	73.13	6.14	70.45	4.29	3.51	0.001**	0.56
SDNN (ms)	46.79	14.96	61.27	18.59	−6.60	0.000***	−1.04
LF (ms^2^)	701.14	119.67	653.29	103.62	3.21	0.003**	0.51
HF (ms^2^)	418.08	175.78	496.14	191.96	−2.88	0.006**	−0.46
LF/HF	1.82	0.94	1.46	0.69	2.98	0.005**	0.47

Air, Room air; EO, C. lanceolata EO; HRV, Heart Rate Variability; BP, Blood Pressure; HR, Heart Rate; SDNN, Standard Deviation of Normal - to - Normal Intervals; LF, Low-Frequency power; HF, High-Frequency power; LF/HF, Ratio of Low-Frequency to High-Frequency power; SBP, Systolic Blood Pressure; DBP, Diastolic Blood Pressure. *N* = 40, values are represented as mean ± SD. Significant differences: ns (not significant); *p < 0.05; **p < 0.01; ***p < 0.001.

### 3.2 Psychological responses

**Figure 6:**
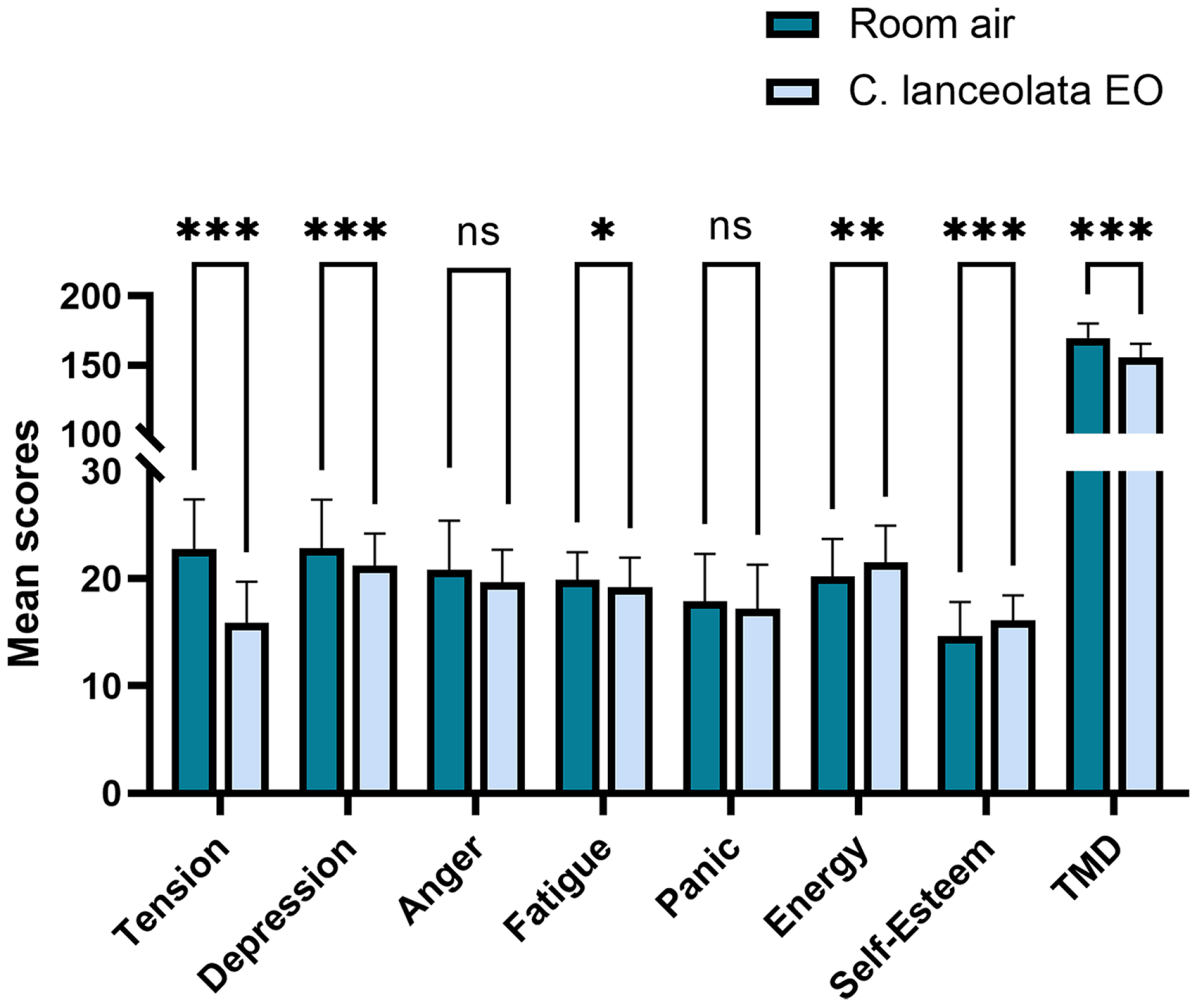
Paired t-test results for POMS scores during inhalation of room air (control) and *C. lanceolata* EO.

Paired-samples t-tests were conducted to determine whether the difference in the mean POMS scores and mean TMD scale scores of the subjects during inhalation of room air (control) and *C. lanceolata* EO was statistically significant (Table 4 and Figure 6). Compared to inhalation of room air (control), subjects who inhaled *C. lanceolata* EOs showed significantly lower scores on negative emotion dimensions, such as “tension,” “depression,” and “fatigue,” and significantly higher scores on positive emotional dimensions such as “energy” and “self-esteem.” Specifically, scores on the “tension” dimension were significantly lower by 6.93 (*p* < 0.001), scores on the “depression” dimension were significantly lower by 1.63 (*p* < 0.001), and scores on the “fatigue” dimension were significantly lower by 0.68 (*p* < 0.05). The scores on the “anger” and “panic” dimensions showed a lower trend, but were not statistically significant: the “anger” score was 1.18 (*p* > 0.05) lower and the “panic” score was 0.70 (*p* > 0.05) lower; the “energy” score, which is a measure of positive emotion, was significantly higher by 1.30 (*p* < 0.01), as did the “self-esteem” score, which was significantly higher by 1.53 (*p* < 0.001). Overall, the TMD score was 13.93 (*p* < 0.001) lower. These results suggest that inhaling *C. lanceolata* EO may have a positive effect on the subjects’ psychological indicators, and it is inferred that it has the potential to improve mood states.

**Table 4:** Paired t-test results of POMS between inhaling Room air (control) and inhaling *C. lanceolata* EO.

Variables	Air		EO		t-test	*p*-value	Cohen’s *d*
	Mean	SD	Mean	SD			
Tension	22.78	4.64	15.85	3.86	9.25	0.000***	1.46
Depression	22.85	4.51	21.23	2.98	3.54	0.001***	0.56
Anger	20.83	4.55	19.65	3.05	1.87	0.069	0.30
Fatigue	19.90	2.54	19.23	2.73	2.04	0.048*	0.32
Panic	17.85	4.46	16.78	4.16	1.34	0.189	0.21
Energy	20.23	3.48	21.53	3.39	−3.37	0.002**	−0.53
Self-Esteem	14.60	3.21	16.13	2.32	−3.96	0.000***	−0.63
TMD	169.38	10.56	155.45	9.74	8.16	0.000***	1.29

Air, Room air; EO, *C. lanceolata* EO; TMD, Total Mood Disturbance, *N* = 40, values are represented as mean ± SD. Significant differences: ns (not significant); *p < 0.05; **p < 0.01; ***p < 0.001.

## 4 Discussion

This study explored the effects of inhaling *C. lanceolata* EO on the physiological and psychological relaxation of university students by using a comprehensive analysis of aromatic compounds in EO, subjects’ electroencephalogram (EEG), HRV, BP, and the POMS. Previous research has shown that the aromatic components in *C. lanceolata* EO can positively impact the physiology and psychology of subjects. The results of this study showed that physiologically, it affects the activity of *α*, *β*, and *θ* waves in EEG and improves HRV and BP indices. Psychologically, subjects’ scores are lower on negative emotions and TMD, while those on positive emotions are higher. Collectively, *C. lanceolata* EO improved the physiological indicators and emotional states of subjects. These findings suggest that inhaling *C. lanceolata* EO has the potential to promote physiological and psychological relaxation in humans.

Regarding the chemical composition, the *C. lanceolata* EO used in this study primarily contains *α*-cedrene (19.04%), cedrol (15.65%), *β*-cedrene (6.86%), and *α*-pinene (0.67%). These components serve as the basis for exploring their physiological and psychological effects. Previous studies have confirmed that cedrol induces obvious relaxation behaviors, demonstrating its relaxation effect at the behavioral level (Kagawa et al., 2003). Inhaling cedrol further affects the central nervous system, producing anti-anxiety and relaxation effects, which verifies its regulatory role in the nervous system through the inhalation pathway (Farimani et al., 2024). Olfactory stimulation by *α*-pinene significantly enhances parasympathetic nervous activity, improves subjective comfort scores, alleviates oxidative stress, and improves memory and mood (Ikei et al., 2016). Building on these findings, the present study hypothesizes that these components may exert similar effects in humans via the inhalation pathway. In conclusion, cedrol and *α*-pinene have anti-anxiety, relaxing, and physiological function-regulating effects. Although direct evidence for the anti-anxiety and relaxation effects of *α*-cedrene and *β*-cedrene is currently limited, the correlation between these compounds and the anti-anxiety and relaxation effects observed in other Cedrus, Cupressus, and Juniperus species suggests a potential role in similar mechanisms of action (Kumar et al., 2023; Lee, 2018; oezek et al., 2021). As common components of plant EOs, *α*-cedrene, cedrol, *β*-cedrene, and *α*-pinene may affect brain excitability via gamma-aminobutyric acid (GABA)-related pathways, producing anti-anxiety and relaxation effects. Olfactory stimulation using relevant EOs can enhance the degree of physiological relaxation, which is consistent with the findings of this study. These results suggest that the volatile components of *C. lanceolata* EO are beneficial to human physical health.

Physiologically, to explore the central nervous response, this study investigated the impact of aroma on EEG activity by examining the effect of *C. lanceolata* EO on brain activity through EEG spectral topographic maps. The results of the experiment showed that there were statistical differences in the *α*, *β*, and *θ* wave power in specific brain regions during inhalation of the two gases. These differences indicate that this EO may play a role in neural regulation by modulating the rhythmic power of multiple brain regions. Specifically, the energy of *α* waves in all brain regions was significantly higher during inhalation of *C. lanceolata* EO (*p* < 0.001). This difference is related to enhanced neuronal synchrony and cortical inhibition and is associated with changes in the EEG activity of the central nervous system during the relaxation response (Kim et al., 2013). Similar studies have reported that after subjects inhaled jasmine fragrance, the power of the *θ* and *α* bands in the frontal lobe increased, accompanied by an improvement in subjective relaxation (Kafaei et al., 2024); inhaling lavender EO increases *α* wave activity, indicating a relaxation effect (Sayorwan et al., 2012); inhaling sweet orange and lavender EOs significantly increases *α* wave activity, indicating a relaxation effect (Je et al., 2021). It was observed that, compared with inhaling room air (control), the *α* wave power in all brain regions was significantly higher during inhalation of *C. lanceolata* EO (*p* < 0.001), which is consistent with previous findings of a significant increase in *α* wave power in aromatherapy studies using various plant EOs. These similar differences in EEG activity suggest that *C. lanceolata* EO may have a neural regulatory mechanism similar to that of other plant EOs, providing a potential relaxation effect.

Compared with room air (control), *β* wave power in the frontal lobe, temporal lobe, and parietal lobe was significantly lower during inhalation of *C. lanceolata* EO (*p* < 0.05). These EEG differences align with prior aromatherapy research. As previously mentioned, a study reported that under relaxation response conditions, *β* wave power in the frontal lobe decreased significantly compared to the control condition. This indicates that the changing trend of *β* wave power can serve as one of the criteria for testing the relaxation effect. A study revealed that when subjects received the *Copaiba* EO intervention, a significant reduction in *β* wave activity occurred in the left frontal region, with this effect linked to a decrease in anxiety (Zhang et al., 2022). Additionally, a study further demonstrated that inhaling *cannabis* EO increased *α* and *θ* wave power while decreasing the relative power of *β* waves, corroborating the association between *β* wave reduction and anti-anxiety states (Gulluni et al., 2018). Temporal lobe *β* activity is usually associated with hypervigilance in cognitive tasks, and the reduction of *β* wave power may reflect decreased temporal cortex excitability, reduce cognitive load, and have a relaxing effect (정한나, 2012). Some scholars have investigated the effects of *Citrus tangerina* EO on brain waves and mood, and the results showed that at the threshold concentration, both *β* wave power was reduced and *θ* wave power was enhanced—presenting a calming characteristic, suggesting that this concentration promotes relaxation by inhibiting cortical excitability (Chandharakool et al., 2020). In conclusion, the significantly lower *β* wave power in the frontal, temporal, and parietal lobes during inhalation of *C. lanceolata* EO is consistent with previous aromatherapy study results showing a relaxation effect. Our research results suggest that *C. lanceolata* EO can shift the subject’s brain from an active, tense state to a relaxed, calm state.

Compared with inhalation of room air (control), *θ* waves were higher overall during inhalation of *C. lanceolata* EO, particularly significant in the frontal and parietal lobes (*p* < 0.05). As previously mentioned, *θ* waves are generally associated with cognition and memory, and also occur in a state of deep relaxation. This more significant change in the frontal lobe and parietal lobe may be related to the functional characteristics and neural connections of these two brain regions (Morgan et al., 2013). As noted earlier, the frontal lobe is responsible for executive functions, emotional regulation, decision-making, etc. The abundant distribution of neurotransmitter receptors makes it more sensitive to the stimulation of aromatic molecules. As a key hub for integrating sensory information, the parietal lobe may have extensive neural fiber projections with the frontal lobe (Andersen and Cui, 2009). The synergistic effect of the two might make the *θ* wave power show more obvious changes under the influence of the *C. lanceolata* EO. Some previous studies have shown that inhalation of the volatile components of *Melia azedarach* flowers can increase theta brainwave activity, playing a positive regulatory role in human physiological and psychological states (Lau et al., 2021); within 0–30 s after inhaling lemon EO, the *θ* and *α* waves in regions such as the frontal lobe were activated, suggesting that short-term inhalation of EOs can affect brain activities; after inhaling *Chrysanthemum indicum* EO, the overall *θ* wave increased, indicating that it may be related to psychophysiological relaxation after inhaling the EO (Kim et al., 2018). In this study, the overall *θ* wave was higher during inhalation of *C. lanceolata* EO, which is consistent with previous research findings and may also indicate that individuals have a tendency toward deep relaxation.

Overall, EEG power shows that the *C. lanceolata* EO positively impacts brain activity by altering the energy of *α*, *β*, and *θ* waves. This finding is similar to previous research on the anti-anxiety effects of EOs based on EEG. The results of this study indicate that, compared to the occipital lobe responsible for visual function, *C. lanceolata* EO has a more significant effect on the frontal lobe related to emotional regulation, the temporal lobe related to olfaction, and the parietal lobe related to cognition. Therefore, based on the functional connectivity of brain regions, it can be inferred that inhaling the scent of this EO may regulate emotional states, induce relaxation responses, and simultaneously promote cognitive processes such as memory and attention.

In addition to EEG power, HRV and BP, which are closely related to the nervous and respiratory systems, are also commonly used to study the mechanisms by which EOs treat emotional disorders (Fung et al., 2021). Inhaling EOs stimulates the olfactory system and exerts influences on the neuroendocrine system, neurotransmitters, and neuromodulators (Angelucci et al., 2014). Through specific physiological pathways, this influence may ultimately induce varying degrees of change in these metrics (Kawai et al., 2020). For instance, studies have shown that inhaling EOs, such as lavender, hinoki, and petitgrain, can cause changes in HRV indicators, reflecting the oils’ ability to reduce stress and improve mood (Wu et al., 2020; Sahhoon et al., 2019; Huang and Capdevila, 2017). Previous studies have shown that the HRV indices of older adults were evaluated during gardening activities while inhaling *Pseudotsuga menziesii* and *Lavandula angustifolia* EOs. Results revealed that normalized low frequency (nLF) and LF/HF power ratios decreased, indicating a relaxing effect of the two EOs on older adults (Chung et al., 2022). Similarly, aromatherapy with lavender and *Damask rose* extracts reduced students’ pre-exam SBP, suggesting diminished stress-related physiological responses (Borzoo et al., 2025). In other studies, when dental patients inhaled *Cymbopogon citratus* EO, significant reductions in SBP, DBP, and HR were observed, implying that *Cymbopogon citratus* EO alleviates anxiety in this population (Maybodi et al., 2025). However, not all aromatic odors reduce stress indicators in humans; for example, one study confirmed that inhalation of *grapefruit* EO significantly increased DBP in healthy subjects through activation of the muscular sympathetic nerve activity (MSNA), suggesting that it may have some physiological pressure-boosting effect (Kawai et al., 2020). There have also been studies investigating the effects of inhaling *rosemary* oil on the sensations of test subjects, as well as its effects on various physiological parameters of the nervous system, which showed significant increases in blood pressure, heart rate, and respiratory rate following inhalation, confirming the stimulatory effects of *rosemary* oil (Sayorwan et al., 2013). The results of the study showed that compared with that during inhalation of room air (control), the stress indicators including HR, LF, and LF/HF ratio were significantly lower (*p* < 0.01) during inhalation of *C. lanceolata* EO, while SDNN and HF were significantly higher (*p* < 0.01) in the same condition. Additionally, both SBP and DBP were significantly lower (*p* < 0.01). These findings align with prior research on EOs-induced autonomic nervous system regulation, collectively suggesting that inhaling *C. lanceolata* EO promotes nervous system self-regulation and induces a relaxation response.

Psychologically, the results of this study show that *C. lanceolata* EO may help improve individual psychological characteristics, which also supports previous research on the benefits of EOs in mood improvement. For instance, a study used POMS to evaluate the psychological state of middle-aged women who inhaled fir EO, the study found significant improvements in subjects’ feelings of comfort, relaxation, natural feeling, and overall mood (Kim et al., 2023). Consistently, other studies have also used POMS to evaluate the mood-improving effects of inhaling EOs. For example, one study found that inhaling the aroma of *Melia azedarach* Linn flowers significantly reduced depression and tension, as well as anger and fatigue, using POMS. These results suggest that *Melia azedarach* Linn flowers or their main compounds have some mood-enhancing effects and can be used in aromatherapy (Lau et al., 2021). Another study used the POMS self-report scale to investigate the effects of exposure to the aroma of pink jasmine flowers on the mood of college students, and the results showed that the indicators of “tension,” “fatigue,” “depression,” “panic,” and TMD were significantly reduced after college students were exposed to the aroma, which supports the idea that “the aroma of pink jasmine flowers is able to improve mood state” (Xiong et al., 2023). Another previous study using the POMS test demonstrated that inhalation of *Citrus junos* Sieb. ex Tanaka oil significantly reduced scores on negative mood subscales such as “tension-anxiety,” “fatigue,” and reduced TMD, confirming its role in alleviating negative emotional stress (Matsumoto et al., 2016). It is also worth noting that another study assessed the effects of two lavender EOs and their chemical components on mood using POMS. The results showed that *Lavandula angustifolia* (high in linalyl acetate and linalool and low in camphor) significantly reduced the negative dimension scores of “tension-anxiety” and “fatigue” as well as the TMD, indicating a significant improvement in the mood state, whereas the reduction in the TMD for *Lavandula spica* (with camphor) was smaller (only 2.1 points) and non-significant. Some samples even showed a reverse effect. This may be due to the fact that the stimulating ingredients (e.g., camphor) in *Lavandula spica* weakened the effect of mood improvement, and even led to a slight discomfort in some subjects. It has been hypothesized that the camphor content (>9%) may antagonize the sedative effect, and therefore this type of EO is not suitable for mood regulation (Tomi et al., 2018).

In other words, not all EOs have an aromatherapeutic effect. Therefore, studies on the specific aromatherapy benefits of various types of EO need to exclude as many confounding factors as possible, while utilizing multiple lines of evidence for validation. In the present study, in addition to the above physiological indicators, the potential function of *C. lanceolata* EO was further demonstrated by POMS. Specifically, compared to that during inhalation of room air, subjects inhaling *C. lanceolata* EO showed significantly lower scores on the negative dimensions of the POMS scale: tension (6.93, *p* < 0.001), depression (1.63, *p* < 0.001), fatigue (0.68, *p* < 0.05), and TMD (13.93, *p* < 0.001). Meanwhile, scores on positive dimensions—such as energy (1.30, *p* < 0.01) and self-esteem (1.53, *p* < 0.001)—were significantly higher. These findings suggest that inhalation of *C. lanceolata* EO may alleviate negative emotions (such as “anxiety,” “depression,” and “fatigue”) and improve mood states significantly; this is hypothesized to be associated with its anxiolytic and relaxing effects.

## 5 Future recommendations

This study had several limitations. Firstly, the EEG cap used in the experiment was made of textile fabric, which may make subjects feel restricted when worn. Secondly, the conductive paste applied may cause discomfort for the subjects, which could induce a stress response and affect the experimental results. For follow-up trials, it is recommended that EEG cap developers replace traditional textile fabrics with medical-grade elastic silicone or breathable nylon-blend materials to enhance the cap’s ductility and reduce pressure on the face and scalp. Developers are also advised to explore dry electrode scalp patches to minimize discomfort caused by conductive paste. In addition, no artificial pressurization was performed during the experiment, but rather room air was used as a control under daily stress conditions, and only the differences with *C. lanceolata* EO intervention and the relaxation effects on university students’ physiology and psychology were analyzed, without exploring its specific stress indicator recovery ability. Future similar studies should consider incorporating artificial stressors and analyzing the intervention effects of EOs under high-stress states, which would enhance the reliability and persuasiveness of the findings. Moreover, the inhalation duration in this experiment was only 5 min. The short exposure time may affect the validity of the conclusions. Future research should design specific habituation tests to record the time it takes for subjects to habituate to the odor of *C. lanceolata* EO, from initial detection until they can no longer perceive it subjectively. On this basis, it should also extend the inhalation cycle to examine whether this habituation process diminishes the positive effects experienced from initially noticing the odor. Finally, the sample size was relatively small (*n* = 40) and consisted solely of university students, resulting in limitations related to a small sample size and a homogeneous population. To deeply explore the benefits of *C. lanceolata* EO, future research should expand the sample size and include subjects from different backgrounds for comparative analysis, thereby enhancing the universality and reliability of the results.

## 6 Conclusion

In summary, significant differences were observed in physiological and psychological indicators during inhalation of *C. lanceolata* EO and room air (control). The main manifestations were as follows: significantly higher *α* and *θ* wave power during inhalation of *C. lanceolata* EO, along with inhibited *β* wave power, characterized by lower alertness and higher relaxation, primarily affecting the frontal, temporal, and parietal regions; sympathetic nerve activity was significantly lower and parasympathetic nerve activity was higher; negative emotions such as tension, depression, and fatigue were lower, and positive emotions such as energy and self-esteem were higher. Based on the definitions and trend implications of the indicators and the results of previous studies, it is hypothesized that it may have a positive relaxation effect on the physiological and psychological state of university students and may have the potential to alleviate stress. This study has made advances in exploring the application value of *C. lanceolata* EO in aromatherapy and provides novel empirical support for integrating natural substances into research on physical and mental regulation and wellbeing.

## Data Availability

The raw data supporting the conclusions of this article will be made available by the authors, without undue reservation.
