# Supplementary material for: Effects of inhaling Cunninghamia lanceolata essential oil on the physiological and psychological relaxation of university students
Source: Front Psychol. 2025 Sep 24;16:1638492. doi: 10.3389/fpsyg.2025.1638492 (PMC12504257; doi:10.3389/fpsyg.2025.1638492)
Supplement: Supplementary file 1 [file Data_Sheet_1.docx]

Supplementary Material

# Supplementary Tables

**Supplementary Table 1.** The main volatile organic compounds of *C. lanceolata EO*

| S.number | Component | Retention time (min) | Formula | Peak area (%) |
| --- | --- | --- | --- | --- |
| 1 | *α*-Cedrene | 33.60 | C₁₅H₂₄ | 19.04 |
| 2 | Cedrol | 38.49 | C₁₅H₂₆O | 15.65 |
| 3 | *β*-Cedrene | 33.81 | C₁₅H₂₄ | 6.86 |
| 4 | n-Hexadecanoic acid | 44.29 | C₁₆H₃₂O₂ | 4.04 |
| 5 | cis-Thujopsene | 34.01 | C₁₅H₂₄ | 3.00 |
| 6 | *β*-Elemene | 32.55 | C₁₅H₂₄ | 2.84 |
| 7 | *α*-Terpinol | 26.60 | C₁₀H₁₈O | 2.63 |
| 8 | *α*-Alaskene | 35.93 | C₁₅H₂₄ | 2.48 |
| 9 | *β*-Selinene | 35.46 | C₁₅H₂₄ | 2.41 |
| 10 | *δ*-Cadinene | 36.043 | C₁₅H₂₄ | 2.34 |
| 11 | *β*-Copaene | 35.193 | C₁₅H₂₄ | 1.78 |
| 12 | Di-epi-α-cedrene-(I) | 32.624 | C₁₅H₂₄ | 1.74 |
| 13 | Cedryl acetate | 41.089 | C₁_7_H_2_₈O_2_ | 1.59 |
| 14 | Octadecanoic acid | 48.172 | C₁_8_H_36_O_2_ | 1.41 |
| 15 | *α*-Selinene | 35.612 | C₁_5_H_24_ | 1.19 |
| 16 | *α*-Pinene | 15.491 | C_10_H_16_ | 0.76 |

**Supplementary Table 2.** Paired t-test results of the EEG power spectrum during inhalation of room air and *C. lanceolata* EO

| Variables(Unit) | Site | Air | | EO | | *t-test* | *p-value* | Cohen’s d |
| --- | --- | --- | --- | --- | --- | --- | --- | --- |
|  |  | *Mean* | *SD* | *Mean* | *SD* |  |  |  |
| Alpha(μv^2^/Hz) | FL | 2.25 | 1.24 | 4.25 | 1.52 | -5.91 | 0.000 *** | -0.93 |
|  | TL | 1.76 | 1.18 | 3.66 | 0.47 | -9.02 | 0.000 *** | -1.43 |
|  | PL | 2.33 | 2.13 | 4.52 | 1.31 | -5.06 | 0.000 *** | -0.80 |
|  | OL | 2.17 | 1.02 | 4.71 | 1.42 | -8.78 | 0.000 *** | -1.39 |
| Beta(μv^2^/Hz) | FL | 1.31 | 0.85 | 0.88 | 0.43 | 3.63 | 0.001 ** | 0.57 |
|  | TL | 1.08 | 0.82 | 0.80 | 0.38 | 2.30 | 0.027 * | 0.36 |
|  | PL | 1.12 | 0.63 | 0.77 | 0.45 | 4.88 | 0.000 *** | 0.77 |
|  | OL | 1.06 | 0.57 | 0.89 | 0.45 | 1.78 | 0.084 | 0.28 |
| Theta(μv^2^/Hz) | FL | 2.66 | 1.06 | 3.67 | 1.67 | -4.26 | 0.000 *** | -0.67 |
|  | TL | 1.98 | 0.80 | 2.21 | 1.99 | -0.70 | 0.490 | -0.11 |
|  | PL | 2.27 | 0.86 | 3.13 | 2.26 | -2.57 | 0.014 * | -0.41 |
|  | OL | 2.89 | 1.07 | 3.03 | 2.86 | -0.34 | 0.732 | -0.05 |

Air, room air; EO, *C. lanceolata* EO; FL, frontal lobe; TL, temporal lobe; PL, parietal lobe; OL, occipital lobe. N = 40, values are represented as mean ± SD. * Significant differences: ns (not significant); **p* < 0.05; ***p* < 0.01; ****p* < 0.001.

**Supplementary** **Table 3.** Paired t-test of HRV and blood pressure (BP) during inhalation of indoor air and *C. lanceolata* EO

| Variables(Unit) | Air | | EO | | *t-test* | *p-value* | Cohen’s d |
| --- | --- | --- | --- | --- | --- | --- | --- |
|  | *Mean* | *SD* | *Mean* | *SD* |  |  |  |
| HR(bmp)  SBP(mmHg) | 87.64 | 13.64 | 73.18 | 6.74 | 8.63 | 0.000 *** | 1.37 |
|  | 116.63 | 7.84 | 112.63 | 6.50 | 3.32 | 0.002 ** | 0.53 |
| DBP(mmHg) | 73.13 | 6.14 | 70.45 | 4.29 | 3.51 | 0.001 ** | 0.56 |
| SDNN(ms) | 46.79 | 14.96 | 61.27 | 18.59 | -6.60 | 0.000 *** | -1.04 |
| LF(ms2) | 701.14 | 119.67 | 653.29 | 103.62 | 3.21 | 0.003 ** | 0.51 |
| HF(ms2) | 418.08 | 175.78 | 496.14 | 191.96 | -2.88 | 0.006 ** | -0.46 |
| LF/HF | 1.82 | 0.94 | 1.46 | 0.69 | 2.98 | 0.005 ** | 0.47 |

Air, Room air; EO, *C. lanceolata* EO; HRV, Heart Rate Variability; BP, Blood Pressure; HR, Heart Rate; SDNN, Standard Deviation of Normal-to-Normal Intervals; LF, Low-Frequency power; HF, High-Frequency power*;* LF/HF, Ratio of Low-Frequency to High-Frequency power; SBP, Systolic Blood Pressure; DBP, Diastolic Blood Pressure. N=40, values are represented as mean ± SD. * Significant differences: ns (not significant); **p* < 0.05; ***p* < 0.01; ****p* < 0.001.

**Supplementary Table 4.** Paired t-test results of POMS during inhalation of room air and *C. lanceolata* EO*.*

| Variables | Air | | EO | | *t-test* | *p-value* | Cohen’s d |
| --- | --- | --- | --- | --- | --- | --- | --- |
|  | *Mean* | *SD* | *Mean* | *SD* |  |  |  |
| Tension | 22.78 | 4.64 | 15.85 | 3.86 | 9.25 | 0.000 *** | 1.46 |
| Depression | 22.85 | 4.51 | 21.23 | 2.98 | 3.54 | 0.001 *** | 0.56 |
| Anger | 20.83 | 4.55 | 19.65 | 3.05 | 1.87 | 0.069 | 0.30 |
| Fatigue | 19.90 | 2.54 | 19.23 | 2.73 | 2.04 | 0.048 * | 0.32 |
| Panic | 17.85 | 4.46 | 16.78 | 4.16 | 1.34 | 0.189 | 0.21 |
| Energy | 20.23 | 3.48 | 21.53 | 3.39 | -3.37 | 0.002 ** | -0.53 |
| Self-Esteem | 14.60 | 3.21 | 16.13 | 2.32 | -3.96 | 0.000 *** | -0.63 |
| TMD | 169.38 | 10.56 | 155.45 | 9.74 | 8.16 | 0.000 *** | 1.29 |

Air, Room air; EO, *C. lanceolata* EO; TMD, Total Mood Disturbance. N=40, values are represented as mean ± SD. * Significant differences: ns (not significant); **p* < 0.05; ***p* < 0.01; ****p* < 0.001.

# Supplementary Figures


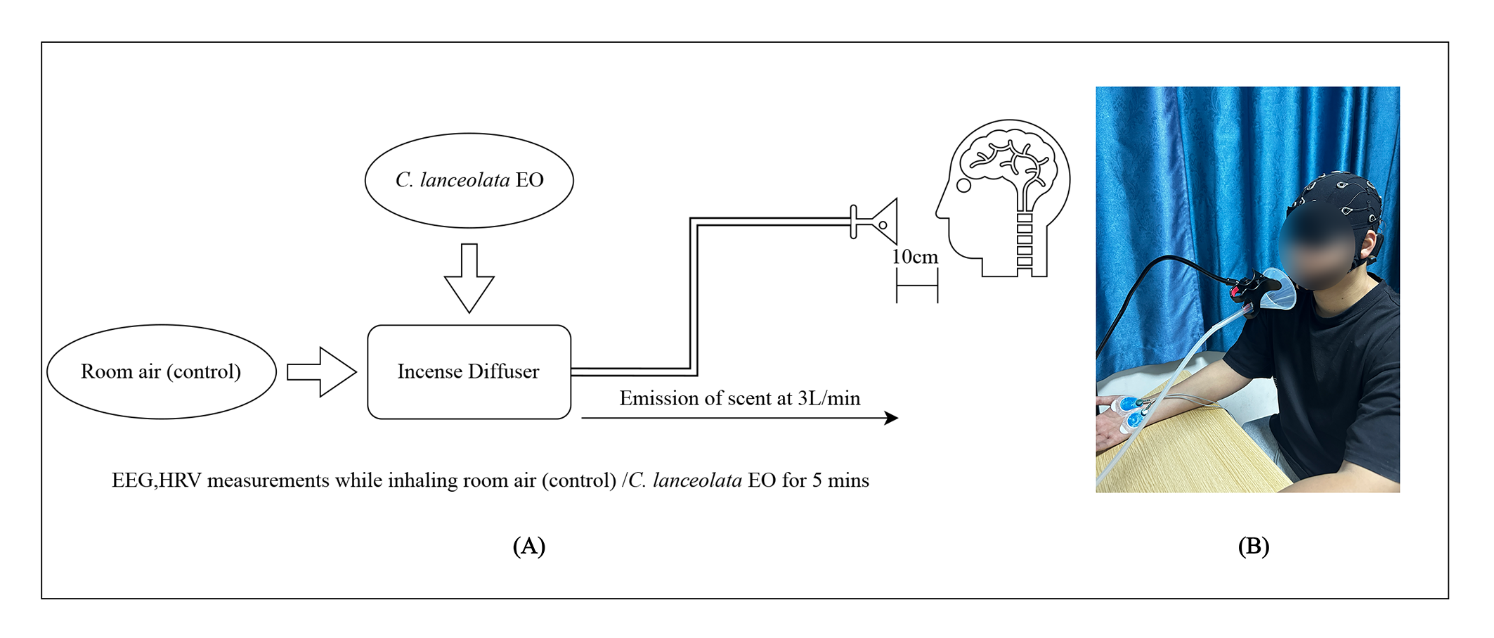


**Supplementary** **Figure 1**. Specification of the experimental setup (A) Aromatherapy diffuser; (B) Experimental scene.

**Figure 1 Alt-Text –** Diagram A illustrates a process involving room air or *C. lanceolata* EO entering an incense diffuser and emitting scent at three liters per minute towards a schematic head at ten centimeters distance. Diagram B depicts a person wearing an EEG cap, inhaling through a mask connected to a tube, with electrodes attached to the arm.


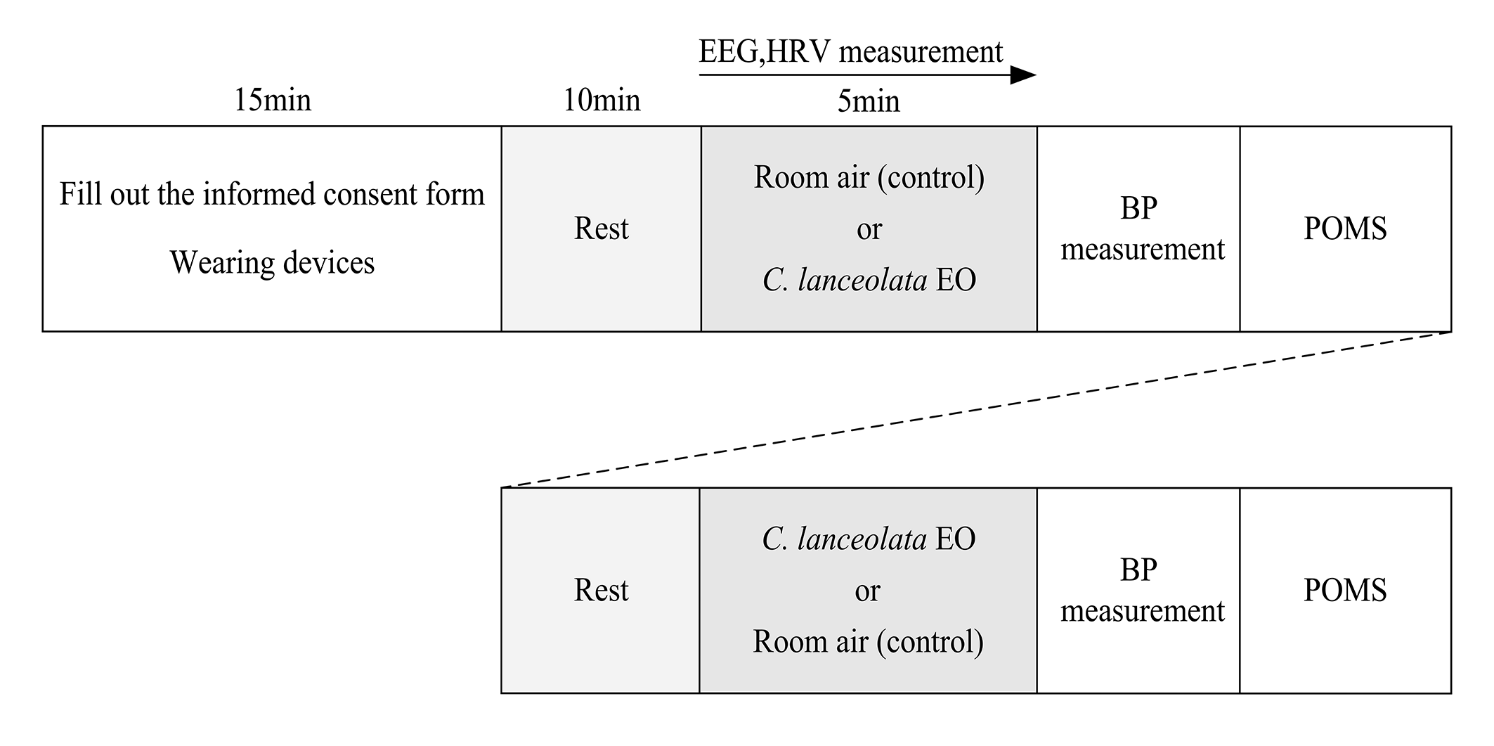


**Supplementary Figure 2.** Experimental procedure. Abbreviations: EEG, Electroencephalogram; HRV, Heart Rate Variability; BP, Blood Pressure; POMS, Profile of Mood States.

**Figure 2 Alt-Text –** Flowchart showing a time sequence for an experiment. The initial 15-minute period includes filling out an informed consent form and wearing devices. Followed by a 10-minute rest and a 5-minute exposure to room air or *C. lanceolata* EO, with EEG and HRV measurements. Ends with blood pressure measurement and POMS assessment. The sequence can alternate between room air and *C. lanceolata* exposure.


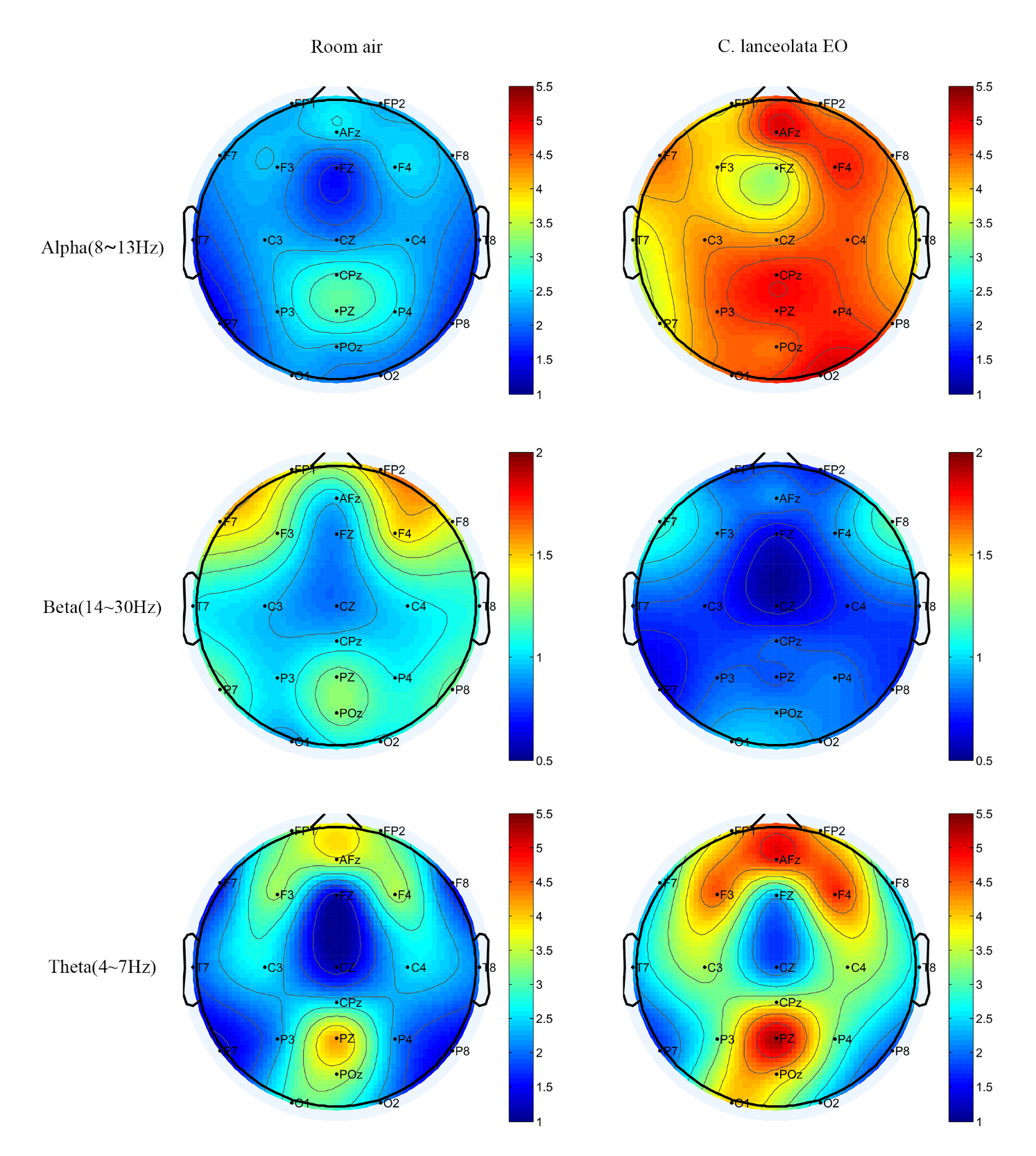


**Supplementary Figure 3.** The t-mapping of EEG power spectrum changes during inhalation of room air and *C. lanceolata* EO.

**Figure 3 Alt-Text –** Color-scaled topographic maps showing brain activity for Alpha, Beta, and Theta frequencies. The left column represents room air conditions, and the right column represents *C. lanceolata* EO conditions. Color scales range from blue (low activity) to red (high activity), with different patterns and intensity levels visible in each map.


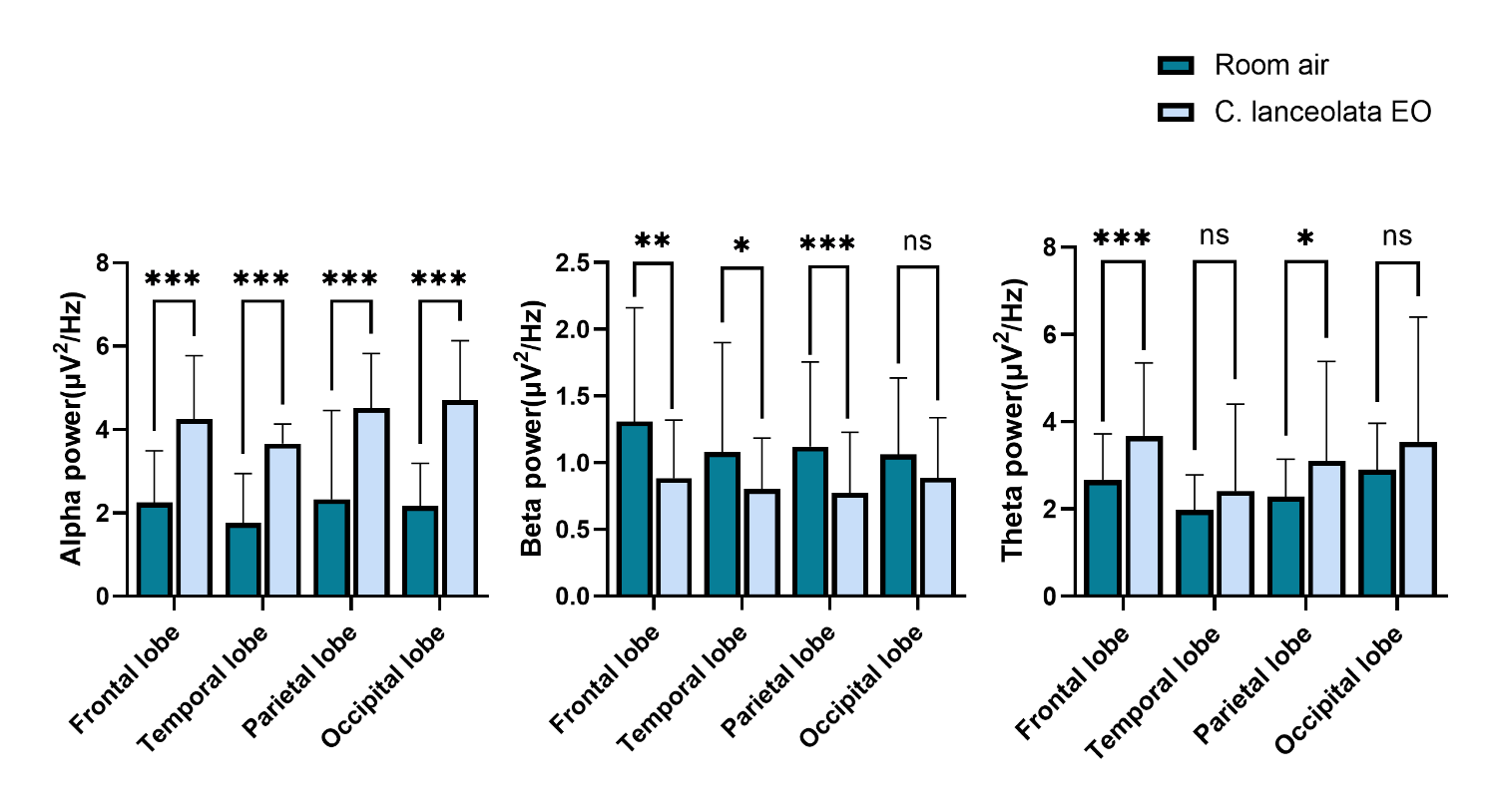


**Supplementary** **Figure 4.** Paired t-test results for EEG power spectrum changes during inhalation of room air and *C. lanceolata* EO. N=40, values are represented as mean ± SD. *Significant differences, (ns, *p* > 0.05; *, *p* < 0.05; **, *p* < 0.01; ***, *p* < 0.001).

**Figure 4 Alt-Text –** Bar graphs comparing alpha, beta, and theta power in four brain lobes between room air and *C. lanceolata* EO conditions. With *C. lanceolata* EO conditions, alpha and theta power are higher, while beta power is lower. Statistical significance is noted with asterisks.


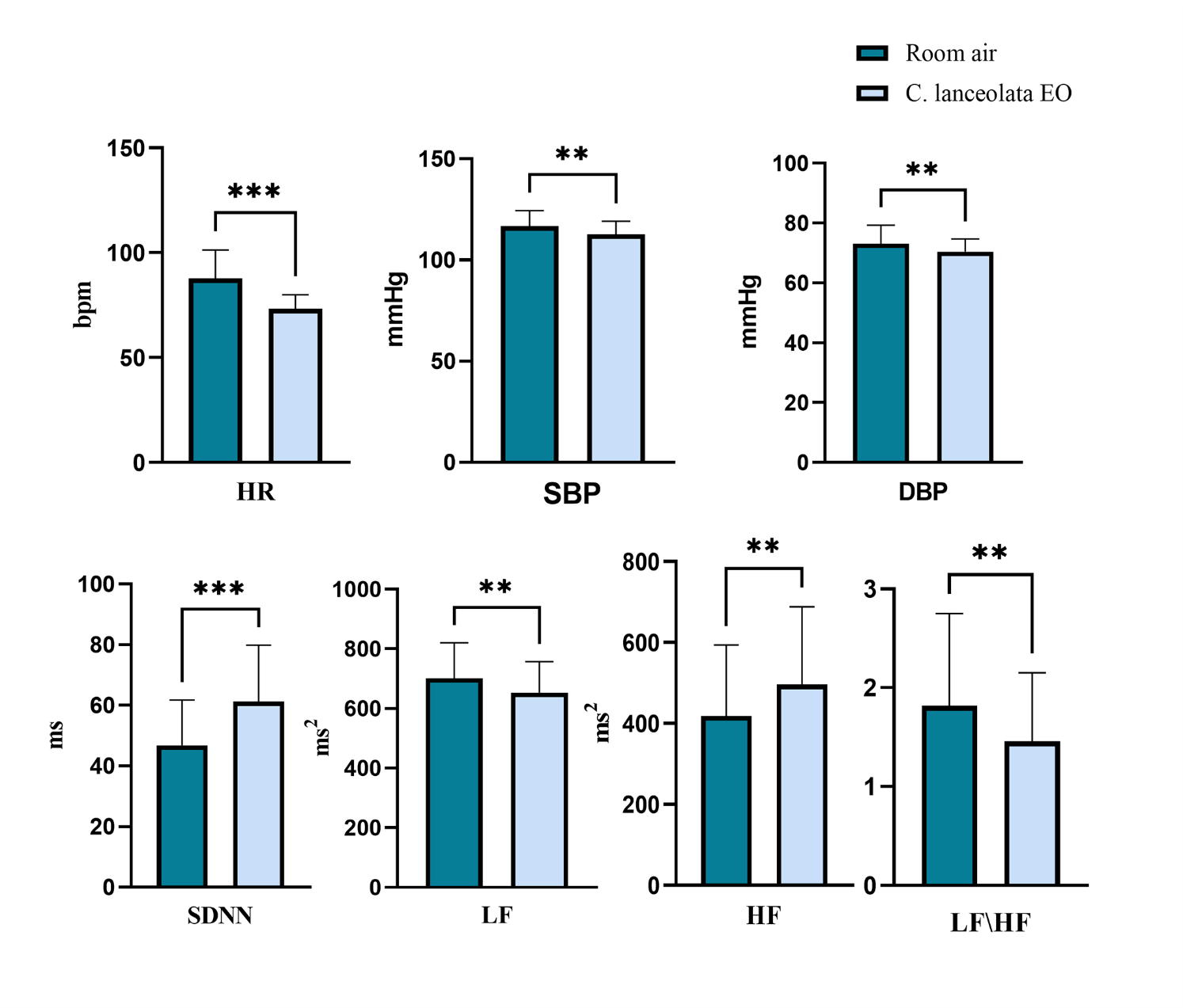


**Supplementary** **Figure 5.** Paired t-test results for HRV and BP during inhalation of room air and *C. lanceolata* EO. Air, Room air; EO, *C. lanceolata* EO; HRV, Heart Rate Variability; BP, Blood Pressure; HR, Heart Rate; SDNN, Standard Deviation of Normal-to-Normal Intervals; LF, Low-Frequency power; HF, High-Frequency power*;* LF/HF, Ratio of Low-Frequency to High-Frequency power*;* SBP, Systolic Blood Pressure*;* DBP, Diastolic Blood Pressure. N=40, values are represented as mean ± SD. *Significant differences, (ns, *p* > 0.05; *, *p* < 0.05; **, *p* < 0.01; ***, *p* < 0.001).

**Figure 5 Alt-Text –** Bar graph comparing physiological metrics between room air and *C. lanceolata* EO conditions. With *C. lanceolata* EO conditions, HR, SBP, DBP, LF, and LF/HF are significantly lower; SDNN and HF are significantly higher. Statistical significance is noted with asterisks.


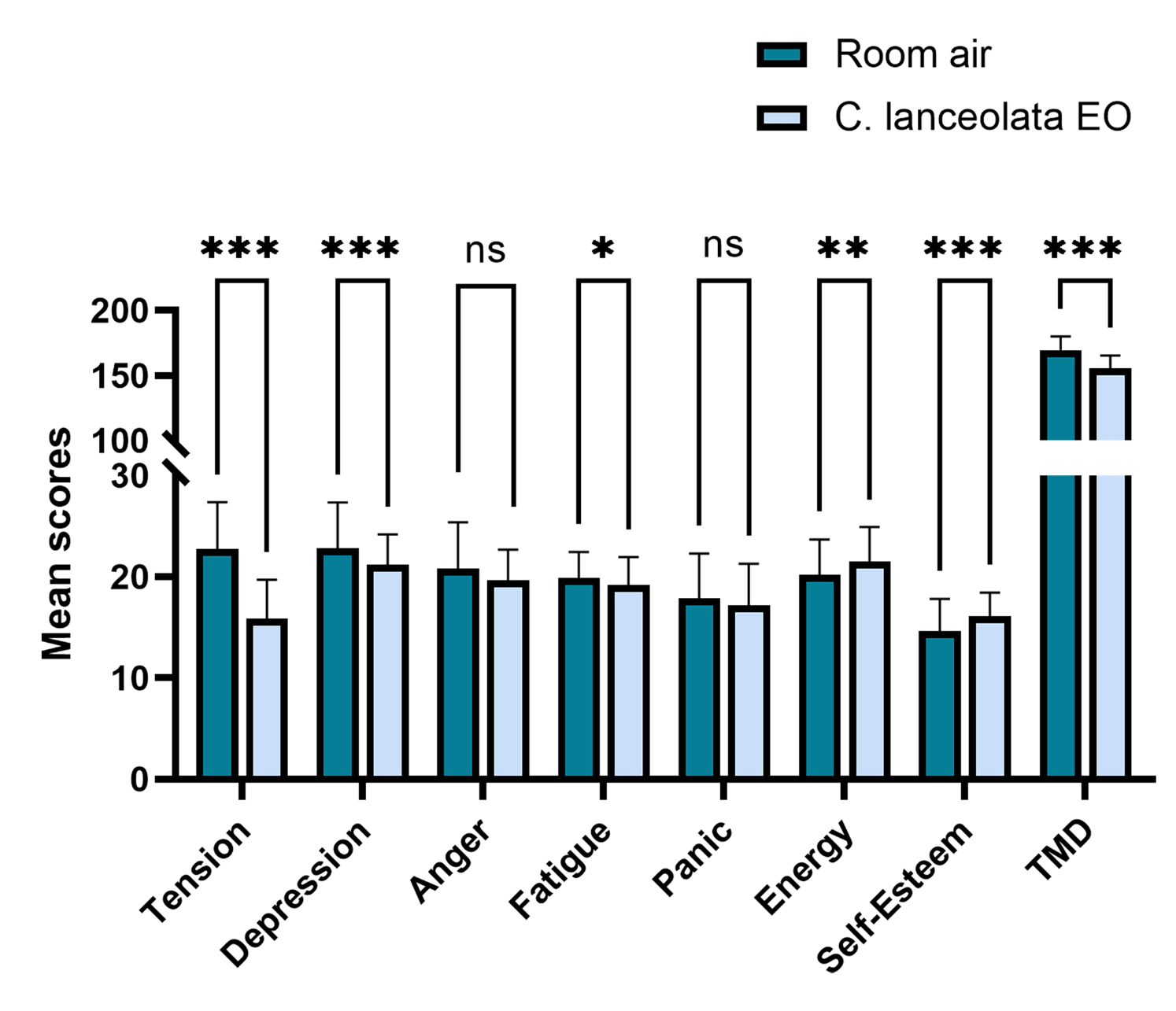


**Supplementary** **Figure 6.** Paired t-test results for POMS scores during inhalation of room air and *C. lanceolata* EO. Abbreviations: TMD, Total Mood Disturbance. N=40, values are represented as mean ± SD. *Significant differences, (ns, *p* > 0.05; *, *p* < 0.05; **, *p* < 0.01; ***, *p* < 0.001).

**Figure 6 Alt-Text –** Bar graph comparing psychology metrics between room air and *C. lanceolata* EO conditions. With *C. lanceolata* EO conditions, tension, depression, fatigue, and Total Mood Disturbance (TMD) are significantly lower; energy, self-esteem are significantly higher. Statistical significance is noted with asterisks.
