# Supplementary material for: Effects of inhaling Cunninghamia lanceolata essential oil on the physiological and psychological relaxation of university students
Source: Front Psychol. 2025 Sep 24;16:1638492. doi: 10.3389/fpsyg.2025.1638492 (PMC12504257; doi:10.3389/fpsyg.2025.1638492)
Supplement: Supplementary file 2 [file Data_Sheet_2.PDF]

Supplementary Material

1 Supplementary Tables

Supplementary Table 1. The main volatile organic compounds of *C. lanceolata EO*

S.number	Component	Retention time (min)	Formula	Peak area (%)
1	α-Cedrene	33.60	$C_{15}H_{24}$	19.04
2	Cedrol	38.49	$C_{15}H_{26}O$	15.65
3	β-Cedrene	33.81	$C_{15}H_{24}$	6.86
4	n-Hexadecanoic acid	44.29	$C_{16}H_{32}O_2$	4.04
5	cis-Thujopsene	34.01	$C_{15}H_{24}$	3.00
6	β-Elemene	32.55	$C_{15}H_{24}$	2.84
7	α-Terpinol	26.60	$C_{10}H_{18}O$	2.63
8	α-Alaskene	35.93	$C_{15}H_{24}$	2.48
9	β-Selinene	35.46	$C_{15}H_{24}$	2.41
10	δ-Cadinene	36.043	$C_{15}H_{24}$	2.34
11	β-Copaene	35.193	$C_{15}H_{24}$	1.78
12	Di-epi-α-cedrene-(I)	32.624	$C_{15}H_{24}$	1.74
13	Cedryl acetate	41.089	$C_{17}H_{28}O_2$	1.59
14	Octadecanoic acid	48.172	$C_{18}H_{36}O_2$	1.41
15	α-Selinene	35.612	$C_{15}H_{24}$	1.19
16	α-Pinene	15.491	$C_{10}H_{16}$	0.76

Supplementary Table 2. Paired t-test results of the EEG power spectrum during inhalation of room air (control) and *C. lanceolata* EO

Variables(Unit)	Site	Air		EO		*t-test*	*p-value*	Cohen's d
		Mean	*SD*	*Mean*	*SD*			
Alpha(μv^2/Hz)	FL	2.25	1.24	4.25	1.52	-5.91	0.000 ***	-0.93
	TL	1.76	1.18	3.66	0.47	-9.02	0.000 ***	-1.43
	PL	2.33	2.13	4.52	1.31	-5.06	0.000 ***	-0.80
	OL	2.17	1.02	4.71	1.42	-8.78	0.000 ***	-1.39
Beta(μv^2/Hz)	FL	1.31	0.85	0.88	0.43	3.63	0.001 **	0.57
	TL	1.08	0.82	0.80	0.38	2.30	0.027 *	0.36
	PL	1.12	0.63	0.77	0.45	4.88	0.000 ***	0.77
	OL	1.06	0.57	0.89	0.45	1.78	0.084	0.28
Theta(μv^2/Hz)	FL	2.66	1.06	3.67	1.67	-4.26	0.000 ***	-0.67
	TL	1.98	0.80	2.21	1.99	-0.697	0.490	-0.11
	PL	2.27	0.86	3.13	2.26	-2.57	0.014 *	-0.41
	OL	2.89	1.07	3.03	2.86	-0.34	0.732	-0.05

Abbreviations: Air, room air; EO, *C*. lanceolata EO; FL, frontal lobe; TL, temporal lobe; PL, parietal lobe; OL, occipital lobe. N = 40; values are represented as mean ± SD. * Significant differences, (ns, $p > 0.05$; *, $p < 0.05$; **, $p < 0.01$; ***, $p < 0.001$).

Supplementary Table 3. Paired t-test of HRV and blood pressure (BP) during inhalation of indoor air (control) and *C. lanceolata* EO

Variables(Unit)	Air		EO		*t-test*	*p-value*	Cohen's d
	Mean	*SD*	*Mean*	*SD*			
HR(bmp)	87.64	13.64	73.18	6.74	8.63	0.000 ***	1.37
SBP(mmHg)	116.63	7.84	112.63	6.50	3.32	0.002 **	0.53
DBP(mmHg)	73.13	6.14	70.45	4.29	3.51	0.001 **	0.56
SDNN(ms)	46.79	14.96	61.27	18.59	-6.60	0.000 ***	-1.04
LF(ms2)	701.14	119.67	653.29	103.62	3.21	0.003 **	0.51
HF(ms2)	418.08	175.78	496.14	191.96	-2.88	0.006 **	-0.46
LF/HF	1.82	0.94	1.46	0.69	2.98	0.005 **	0.47

Abbreviations: Air, Room air; EO, *C. lanceolata* EO; HRV, Heart Rate Variability; BP, Blood Pressure; HR, Heart Rate; SDNN, Standard Deviation of Normal - to - Normal Intervals; LF, Low - Frequency power; HF, High - Frequency power; LF/HF, Ratio of Low - Frequency to High - Frequency power; SBP, Systolic Blood Pressure; DBP, Diastolic Blood Pressure. N=40, values are represented as mean ± SD. * Significant differences, (ns, $p > 0.05$; *, $p < 0.05$; **, $p < 0.01$; ***, $p < 0.001$).

Supplementary Table 4. Paired t-test results of POMS between inhaling Room air (control) and inhaling *C. lanceolata* EO.

Variables	Air		EO		*t-test*	*p-value*	Cohen’s d
	Mean	*SD*	*Mean*	*SD*			
Tension	22.78	4.64	15.85	3.86	9.25	0.000 ***	1.46
Depression	22.85	4.51	21.23	2.98	3.54	0.001 ***	0.56
Anger	20.83	4.55	19.65	3.05	1.87	0.069	0.30
Fatigue	19.90	2.54	19.23	2.73	2.04	0.048 *	0.32
Panic	17.85	4.46	16.78	4.16	1.34	0.189	0.21
Energy	20.23	3.48	21.53	3.39	-3.37	0.002 **	-0.53
Self-Esteem	14.60	3.21	16.13	2.32	-3.96	0.000 ***	-0.63
TMD	169.38	10.56	155.45	9.74	8.16	0.000 ***	1.29

Abbreviations: Air, Room air; EO, *C. lanceolata* EO ; TMD,Total Mood Disturbance, N=40, values are represented as mean ± SD. * Significant differences, (ns, $p > 0.05$; *, $p < 0.05$; **, $p < 0.01$; ***, $p < 0.001$).

2 Supplementary Figures

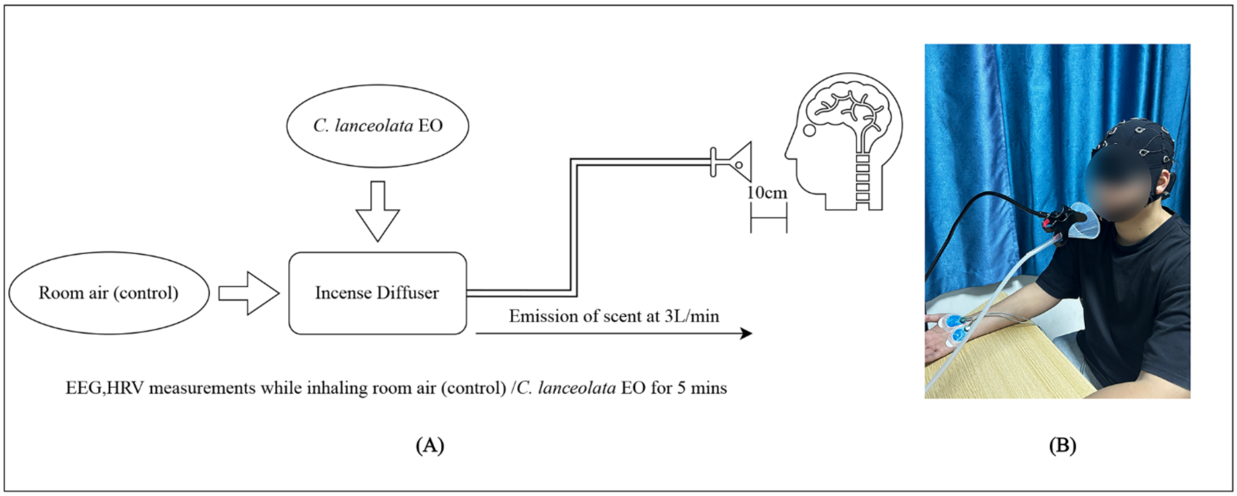

Supplementary Figure 1. Specification of the experimental. (A) Aromatherapy diffuser; (B) Experimental scene.

Describe-content-images: Figure 1 illustrates the apparatus and practical operation of the *C. lanceolata* EO inhalation experiment. Schematic diagram A illustrates the use of an incense diffuser to atomize room air or *C. lanceolata* EO at a rate of 3 L/min, 10 cm from the subject's nose. Electroencephalogram (EEG) and heart rate variability (HRV) are measured during the five-minute inhalation period. Picture B is a photo of the experimental setup, showing the participant wearing an electrode cap and other equipment while undergoing the inhalation experiment.

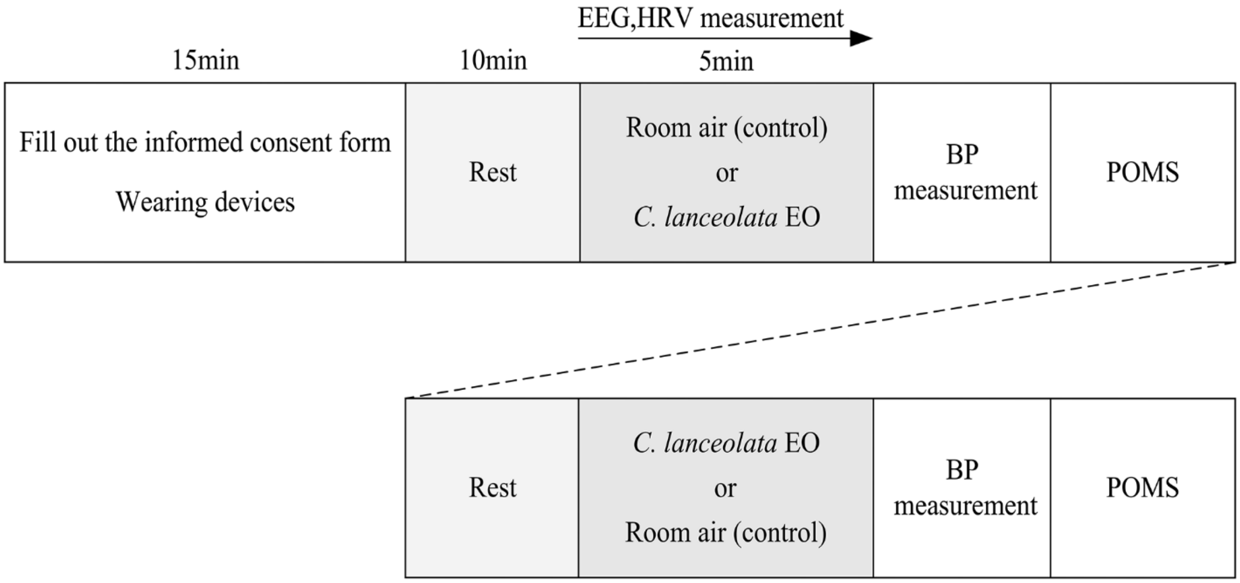

Supplementary Figure 2. Experimental procedure. Abbreviations: EEG, Electroencephalogram; HRV, Heart Rate Variability; BP, Blood Pressure; POMS, Profile of Mood States.

Describe-content-images: Figure 2 illustrates the experimental procedure. First, participants spent 15 minutes filling out the informed consent form and putting on the equipment. Then, they rested for 10 minutes. Afterwards, in terms of sequence, approximately half of the participants first inhaled room air (control) for 5 minutes, and then inhaled *C. lanceolata* essential oil for 5 minutes. During this time, electroencephalograms (EEG) and heart rate variability (HRV) were measured. Then, blood pressure was measured, and participants completed the Profile of Mood States (POMS) questionnaire. For the remaining participants, the order of the inhaled gases was reversed to eliminate order effects, while the other steps remain the same.

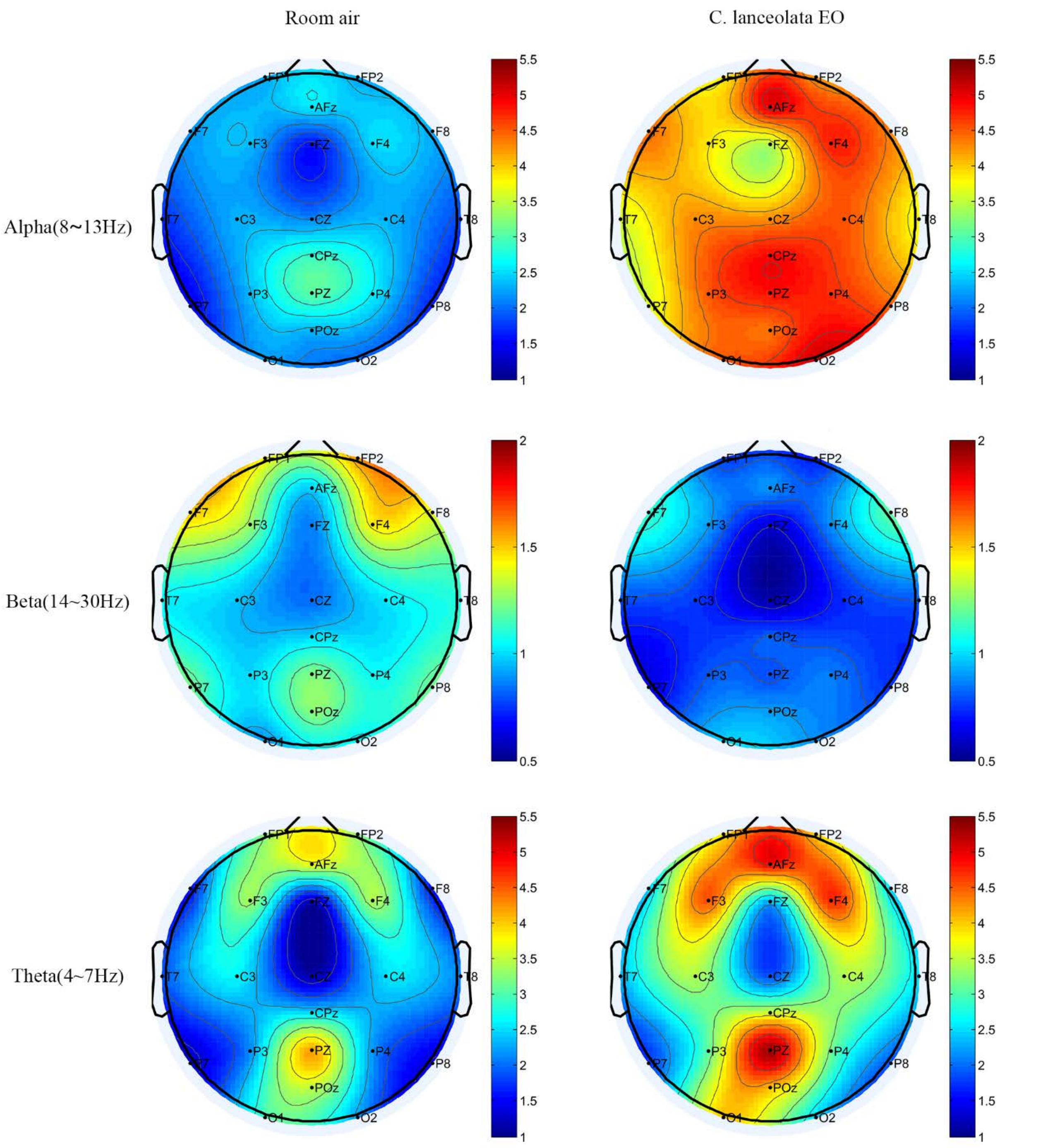

Supplementary Figure 3. The t-mapping of EEG power spectrum changes during inhalation of room air and *C. lanceolata* EO.

Describe-content-images: Figure 3 illustrates the t-map of changes in the power spectrum of electroencephalogram (EEG) during inhalation of room air or *C. lanceolata* EO. It presents the distribution of the three brain wave frequency bands, α (8 – 13 Hz), β (14 – 30 Hz), and θ (4 – 7 Hz), under different inhalation conditions.

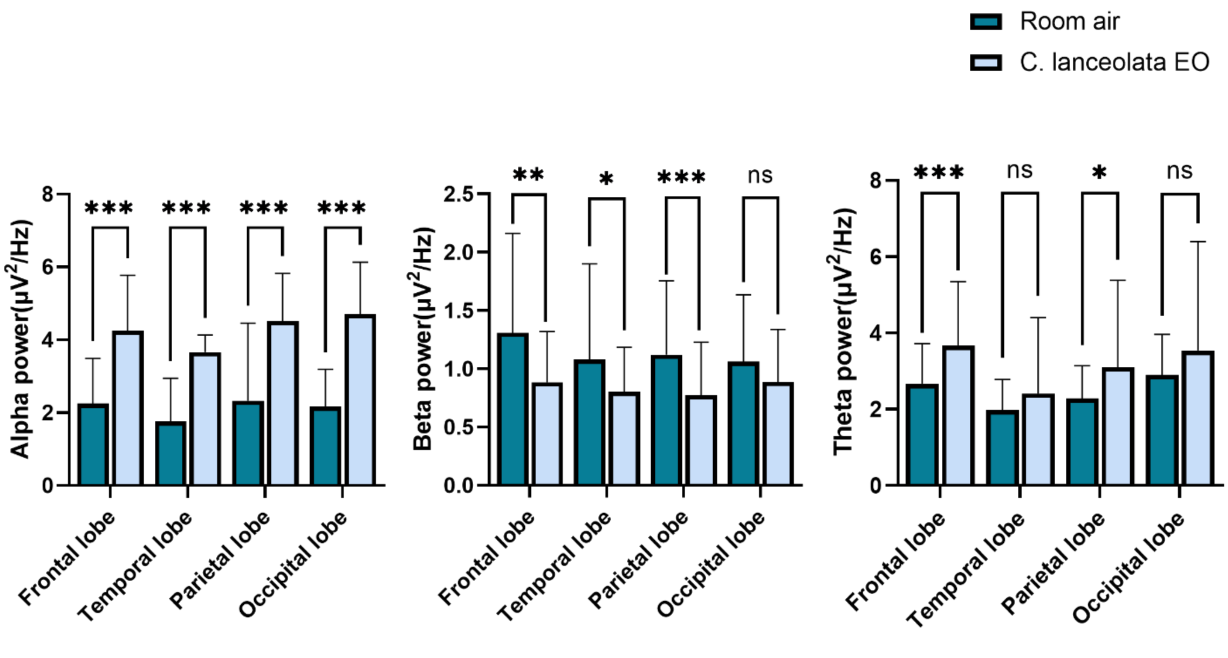

Supplementary Figure 4. Paired t-test results for EEG power spectrum changes during inhalation of room air and *C. lanceolata* EO. N=40, values are represented as mean ± SD. *Significant differences, (ns, $p > 0.05$; *, $p < 0.05$; **, $p < 0.01$; ***, $p < 0.001$).

Describe-content-images: Figure 4 illustrates the results of a paired t-test for changes in electroencephalogram (EEG) power spectra when inhaling room air and *C. lanceolata* EO (N = 40). The power changes in the frontal, temporal, parietal, and occipital lobes in the α, β, and θ frequency bands are shown, presented as mean ± standard error, with asterisks indicating significant differences (ns indicates $p > 0.05$, * indicates $p < 0.05$, ** indicates $p < 0.01$, and *** indicates $p < 0.001$).

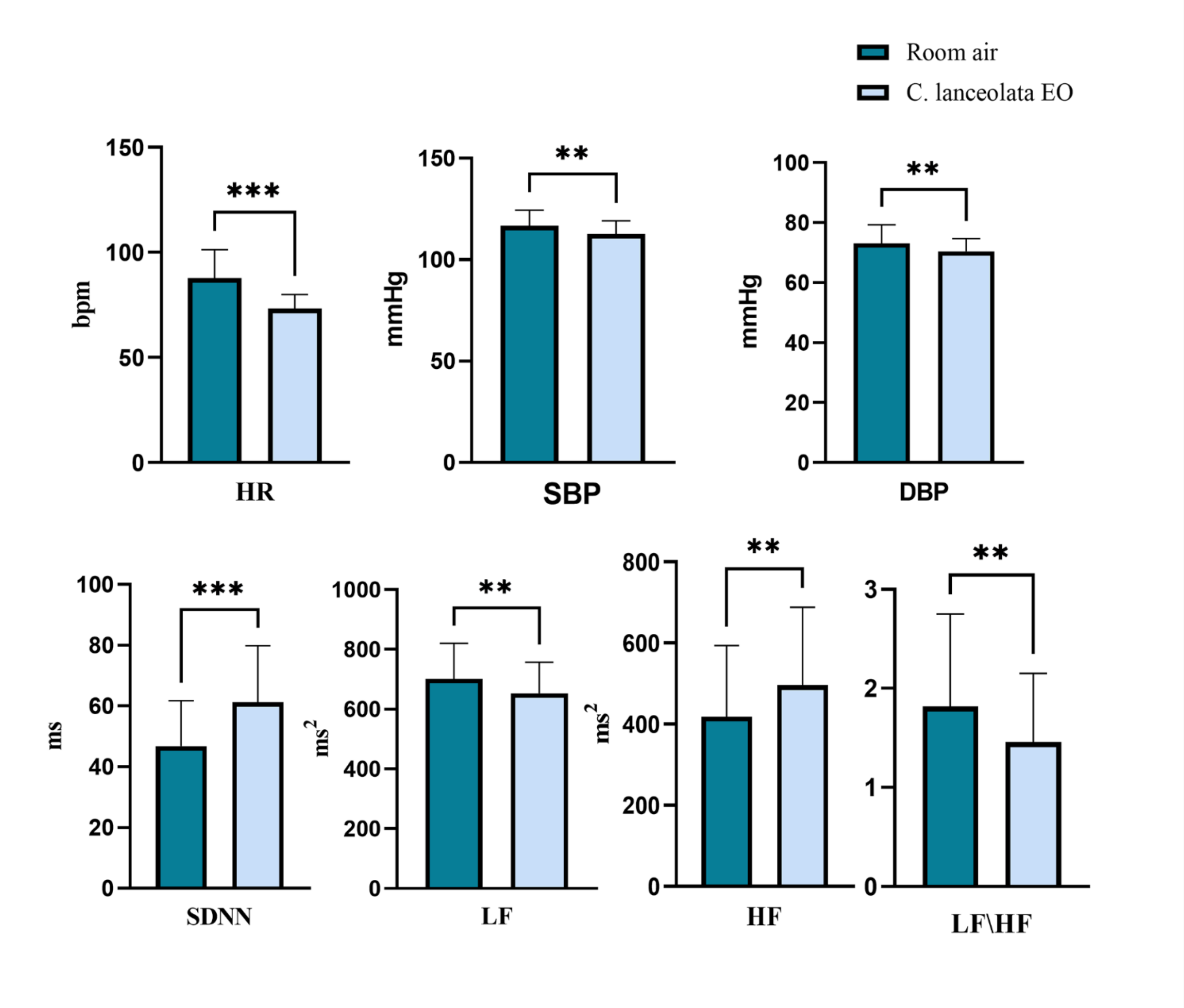

Supplementary Figure 5. Paired t-test results for heart rate variability (HRV) and blood pressure (BP) during inhalation of room air (control) and *C. lanceolata* EO. Abbreviations: SBP, systolic blood pressure; DBP, diastolic blood pressure; N=40, values are represented as mean ± SD. *Significant differences, (ns, $p > 0.05$; *, $p < 0.05$; **, $p < 0.01$; ***, $p < 0.001$).

Describe-content-images: Figure 5 illustrates the results of paired t-tests for heart rate variability (HRV) and blood pressure (BP) when inhaling room air (control) and *C. lanceolata* EO (N = 40), with values expressed as mean ± standard error, and significant differences marked with asterisks (ns indicates $p > 0.05$, * indicates $p < 0.05$, ** indicates $p < 0.01$, and *** indicates $p < 0.001$).

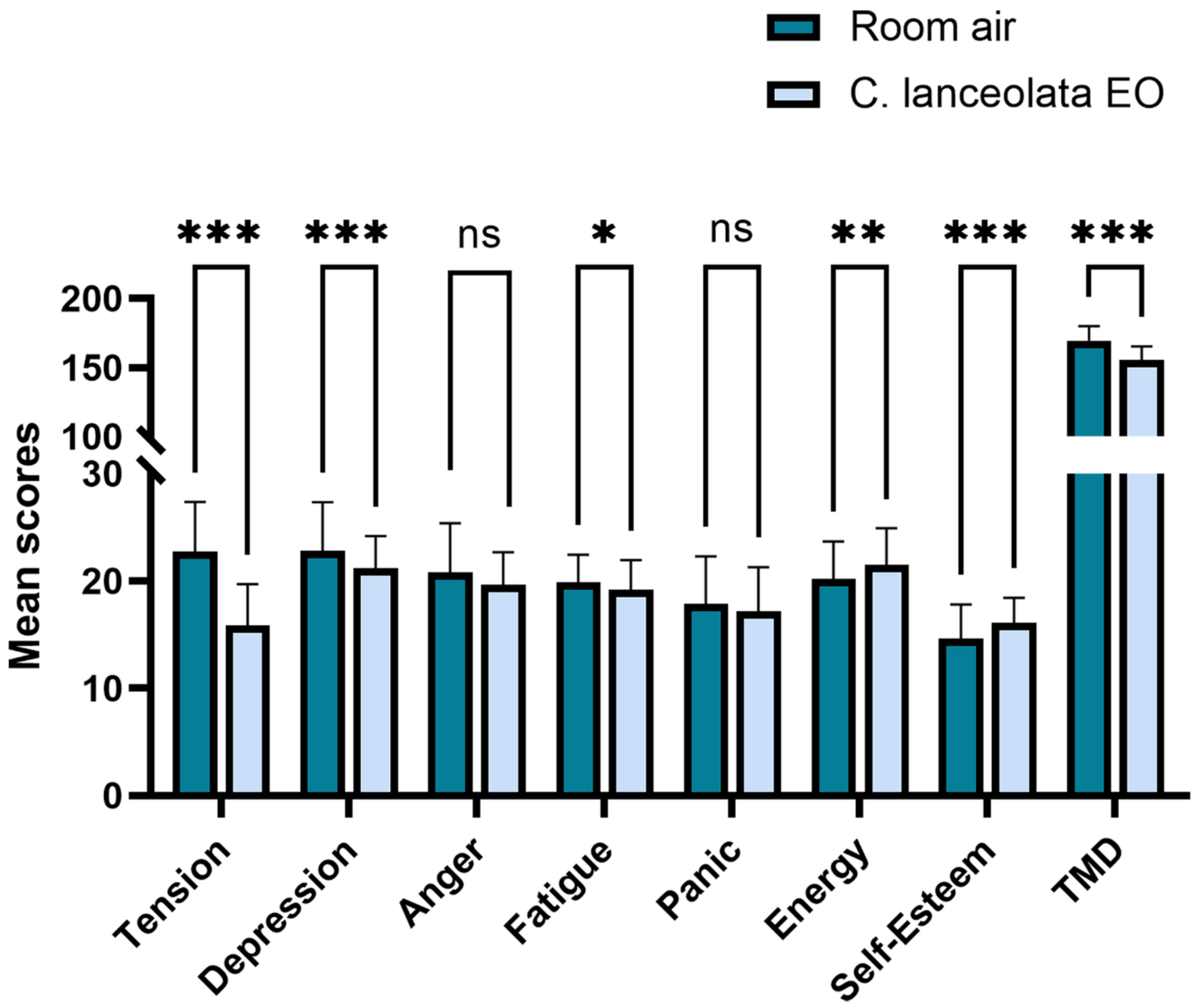

Supplementary Figure 6. Paired t-test results for POMS scores during inhalation of room air (control) and *C. lanceolata* EO. Abbreviations: TMD, Total Mood Disturbance; N=40, values are represented as mean ± SD. *Significant differences, (ns, $p > 0.05$; *, $p < 0.05$; **, $p < 0.01$; ***, $p < 0.001$).

Describe-content-images: Figure 6 c the results of a paired t-test comparing the scores on the Profile of Mood States (POMS) between inhaling room air (control) and *C. lanceolata* EO (N = 40). The figure presents the average scores for dimensions such as Tension, Depression, Anger, Fatigue, Panic, Energy, Self-Esteem, and Total Mood Disturbance (TMD), expressed as mean ± standard error. Significant differences are marked with asterisks (ns indicates $p > 0.05$, * indicates $p < 0.05$, ** indicates $p < 0.01$, and *** indicates $p < 0.001$).
